# Nucleic Acid Amplification Circuit‐Based Hydrogel (NACH) Assay for One‐Step Detection of Metastatic Gastric Cancer‐Derived Exosomal miRNA

**DOI:** 10.1002/advs.202407621

**Published:** 2024-09-23

**Authors:** Seung Beom Seo, Jaewoo Lim, Kyujung Kim, Inhee Maeng, Hyun Wook Rho, Hye Young Son, Eunjung Kim, Eunji Jang, Taejoon Kang, Juyeon Jung, Seung Jae Oh, Yong‐Min Huh, Eun‐Kyung Lim

**Affiliations:** ^1^ Bionanotechnology Research Center Korea Research Institute of Bioscience and Biotechnology (KRIBB) Daejeon 34141 Republic of Korea; ^2^ Department of Cogno‐Mechatronics Engineering Pusan National University Pusan 46241 Republic of Korea; ^3^ Medical Device Development Center Osong Medical Innovation Foundation 123, Osongsaengmyeong‐ro Chungcheongbuk‐do 28160 Republic of Korea; ^4^ YUHS‐KRIBB Medical Convergence Research Institute Yonsei University Seoul 03772 Republic of Korea; ^5^ Department of Radiology Yonsei University Seoul 03772 Republic of Korea; ^6^ Department of Bioengineering & Nano‐bioengineering Research Center for Bio Materials and Process Development Incheon National University Incheon 22012 Republic of Korea; ^7^ Division of Bioengineering Incheon National University Incheon 22012 Republic of Korea; ^8^ MediBio‐Informatics Research Center Novomics Co., Ltd. Seoul 07217 Republic of Korea; ^9^ School of Pharmacy Sungkyunkwan University Suwon 16419 Republic of Korea; ^10^ Department of Nanobiotechnology KRIBB School of Biotechnology UST Daejeon 34113 Republic of Korea; ^11^ Department of Biochemistry & Molecular Biology College of Medicine Yonsei University Seoul 03722 Republic of Korea

**Keywords:** exosomal miRNA, gastric cancer, hydrogel assay, nucleic acid amplification circuit, portable fluorometer

## Abstract

Gastric cancer (GC) is recognized as the fifth most prevalent malignant tumor worldwide. It is characterized by diverse clinical symptoms, treatment responses, and prognoses. In GC prognosis, the promotion of epithelial–mesenchymal transition (EMT) fosters cancer cell invasion and metastasis, thereby triggering the dissemination of tumor cells. This study proposes a nucleic acid amplification circuit‐based hydrogel (NACH) assay for identifying exosomal miRNA derived from metastatic GC. The NACH assay employs the rolling circle amplification method and targets miRNA‐21, a tumor‐related oncogene, and miRNA‐99a, which promotes EMT. Specific amplification probes for each target are immobilized within the hydrogel, enabling a streamlined, one‐step amplification reaction. The NACH assay exhibits a detection limit of 1 fm for miRNA‐21 and miRNA‐99a, thereby enabling rapid and highly sensitive on‐site detection. Performance evaluation using exosomal miRNA extracted from cell culture media, mouse plasma, and human plasma revealed fluorescence intensity patterns similar to those obtained in qRT‐PCR. Furthermore, deploying a custom‐developed portable fluorometer for the NACH assay allows for diagnostic performance assessment and point‐of‐care testing using clinical samples from GC patients. These findings emphasize the potential of the NACH assay to be used as a robust tool for the genetic diagnosis of GC based on exosome detection.

## Graphical abstract

The Nucleic Acid Amplification Circuit‐Based Hydrogel (NACH) assay is developed to detect metastatic gastric cancer (GC)‐related exosomal miRNAs biomarkers. This assay can analyze exosomal miRNA expression levels in a one‐step, and its performance has been demonstrated using blood samples from GC patients at both early and metastatic stages. The NACH assay shows potential as a diagnostic tool for liquid biospy.

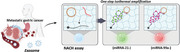

## 1 Introduction

Despite continuous improvement in diagnosis and treatment within the field of oncology, gastric cancer (GC) remains the fifth most prevalent malignant tumor and the fourth leading cause of cancer‐related deaths, according to the World Health Organization (WHO).^[^ ^1^ ^]^ Notably, more than 1 million cases were diagnosed globally annually in 2020, with 770000 GC patients dying each year.^[^ ^2^ ^]^ Importantly, GC is a heterogeneous tumor characterized by complex genetic and epigenetic changes. As a result, it is characterized by various clinical symptoms, treatment responses, and prognoses. GC has been sub‐classified into subtypes with distinctive biological characteristics related to clinical outcomes, using classifiers developed by The Cancer Genome Atlas and the Asian Cancer Research Group (ACRG).^[^ ^3^ ^]^ Although the mechanisms underlying the heterogeneity of GC are not fully understood, it has been reported that patients with the epithelial–mesenchymal transition (EMT) subtype have a higher risk of recurrence and lower survival rates.^[^ ^4^ ^]^ Herein, EMT is a process that plays a crucial role in pathological processes, including embryonic development, tumor formation, and progression. EMT promotion can be induced by the overexpression of transcription factors functioning downstream of various signaling pathways, such as TGF‐β, Wnt/β‐catenin, and Notch, which are known to cross‐talk with each other.^[^ ^5^ ^]^ This phenomenon facilitates cancer cell stemness, invasion, and metastasis, enabling the systemic dissemination of tumor cells. miRNAs are small non‐coding RNAs (≈25 nucleotides in length) that bind to the 3′ UTR of mRNA to regulate gene expression. miRNAs dysregulation is closely associated with cancer initiation, development, and metastasis.^[^ ^6^ ^]^ Specifically, miRNA‐21 acts as an oncogene by downregulating tumor suppressor genes like Phosphatase and tensin homolog (PTEN), leading to increased tumor cell proliferation, apoptosis resistance, and invasion. It also induces mesothelial‐to‐mesenchymal transition (MMT) in peritoneal mesothelial cells, contributing to peritoneal metastasis.^[^ ^7^ ^]^ miRNA‐99a, in addition, promotes TGF‐β‐induced EMT, enhancing cancer cell migration and invasion. Its overexpression is notably associated with signet‐ring cell carcinoma (SRC), a subtype of gastric cancer, and correlates with poor prognosis due to its role in promoting cell proliferation.^[^ ^8^ ^]^ Notably, miRNAs present within exosomes as crucial mediators of cell‐cell communication, facilitating interactions between cancer cells and normal cells.^[^ ^9^ ^]^ Therefore, the detection of exosomal miRNAs is emerging as an ideal biomarker for cancer diagnosis and prognosis monitoring.^[^ ^10^ ^]^ Recent studies have reported the development of systems capable of rapidly and sensitively detecting exosomal miRNAs, but the rapid analysis of miRNAs remains a challenge due to their short length and high sequence similarity.^[^ ^11^ ^]^ Traditionally, miRNAs have been detected using quantitative real‐time polymerase chain reaction (qRT‐PCR) and microarrays.^[^ ^12^ ^]^ However, these methods have inherent limitations, including time intensiveness, high cost, and complexity.^[^ ^13^ ^]^ In recent years, efforts to address these limitations have focused on researching enzyme‐ or non‐enzyme‐based amplification reactions. These include rolling circle amplification (RCA), loop‐mediated isothermal amplification (LAMP), exponential amplification reaction (EXPAR), catalytic hairpin assembly, and hybridization chain reaction (HCR).^[^ ^14^ ^]^ Among these, RCA has the advantages, such as high sensitivity and efficient amplification of specific sequences. However, it is associated with a limitation, i.e., increased background signal due to non‐specific amplification.^[^ ^15^ ^]^ To overcome the drawbacks associated with the use of RCA, 3D structured hydrogel was introduced. Hydrogel‐based detection systems offer economic feasibility in manufacturing and are highly regarded in the biomedical field for their affordability and ability to enable high specificity detection through their 3D structure.^[^ ^16^ ^]^ Herein, we have developed a nucleic acid amplification circuit‐based hydrogel (NACH) assay for the exosomal miRNA derived from metastatic GC. Initially, the target miRNA is amplified through the RCA reaction, followed by the binding of a fluorescent‐labeled ssDNA to the amplification product, resulting in a fluorescent signal. This enables the sensitive detection of target miRNA even at low concentrations. The hydrogel offers a large surface area for effectively immobilizing DNA probes that selectively bind to target miRNAs, thereby enhancing the assay's environmental stability and detection performance. Furthermore, we validated the NACH assay's performance using clinical samples from GC patients (10 healthy individuals, 20 patients with early‐stage disease, and 20 patients with progressive‐stage disease). Our findings demonstrate that analyzing miRNAs associated with GC promotion and EMT serves as an effective tool for cancer diagnosis and prognosis monitoring.

## 2 Results and Discussion

### 2.1 Workflow of NACH Assay

**Figure 1:**
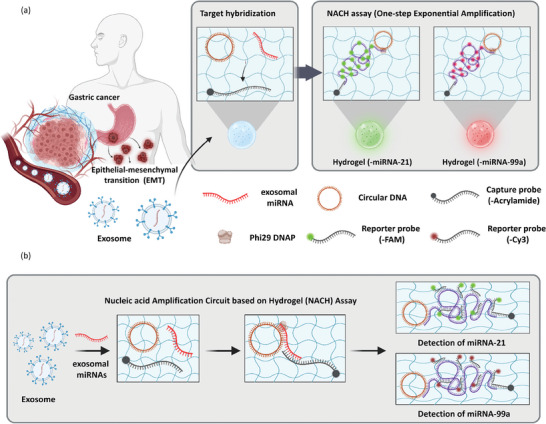
Concept and design of the nucleic acid amplification circuit‐based hydrogel (NACH) assay. a) Schematic diagram illustrating the detection of exosomal miRNAs that induce EMT promotion in gastric cancer patients. Detection of miRNA‐21 and miRNA‐99a in the NACH assay. b) Schematic overview of miRNA detection using the NACH assay. Isothermal amplification is performed through the binding of miRNA‐21 and miRNA‐99a to the circular DNA (circularized padlock probe) and the capture probe. The amplification product exhibits a fluorescent signal upon binding to the reporter probe (miRNA‐21: Fluorescein amidite (FAM) probe; miRNA‐99a: Cyanine3 (Cy3) probe).

The strategy for NACH‐mediated one‐step detection of GC‐derived exosomal miRNAs is depicted in **Figure** **1**. The NACH assay is an enzymatic cascade reaction that utilizes the RCA system. It is specifically designed to achieve exponential amplification of the probe, mediated by the target miRNA. As shown in Figure 1a, exosomal miRNAs isolated from the plasma of GC patients were used in the NACH assay. Notably, our goal is to ascertain the invasion and metastasis of cancer cells into adjacent tissues by detecting tumor markers, i.e., miRNAs, that are involved in EMT. NACH facilitates one‐step detection through the hybridization of circular DNA, target miRNA, and capture probe into the hydrogel (Figure 1b). To immobilize the detection probes within the hydrogel, an acrylamide group was attached to the 3′ end of the capture probe, which was then allowed to interact with polyethylene glycol diacrylate (PEG‐DA), a hydrogel precursor; this enabled the formation of the hydrogel. The system was designed in such a way that half (11 bp) of the target miRNA (22 bp) binds to the capture probe within the hydrogel, while the other half (11 bp) binds to the circular DNA, thereby initiating the detection reaction inside the hydrogel (Figure S1, Supporting Information). Total RNA was extracted from plasma‐derived exosomes, with the target miRNA serving as the initiator for this NACH assay. The reporter probes for miRNA‐21 and miRNA‐99a were designed to bind to the amplification products generated by RCA. The reporter probe used for miRNA‐21 detection was 5′ labeled with fluorescein phosphoramidite (FAM), while the reporter probe used for miRNA‐99a detection was 5′ labeled with cyanine 3 (Cy3). Washing step is an essential process that enables the specific identification of fluorescent signals for the detection of target miRNAs. Designed padlock probe, capture probe, and circular DNA were validated for off‐target effects, cross‐reactivity, and secondary structure formation using nucleic acid analysis software (NUPACK Software), ensuring optimal hybridization and amplification efficiency (Figure S2 and Table S1, Supporting Information). Through NUPACK analysis, which allows for secondary structure prediction, sequence interaction analysis, and free energy calculation, we analyzed the binding performance according to each probe sequence length and selected probe sequences applicable within the system (Figure S3, Supporting Information). All probes and PEG‐DA were placed ins a cylindrical shaped‐mold created through soft lithography using a 3D printer and polydimethylsiloxane (PDMS), and then irradiated with UV light (λ_ex_ = 365 nm) to generate the NACH platform, which was subsequently was washed with deionized water before use.

### 2.2 NACH for Exosomal miRNA Detection

**Figure 2:**
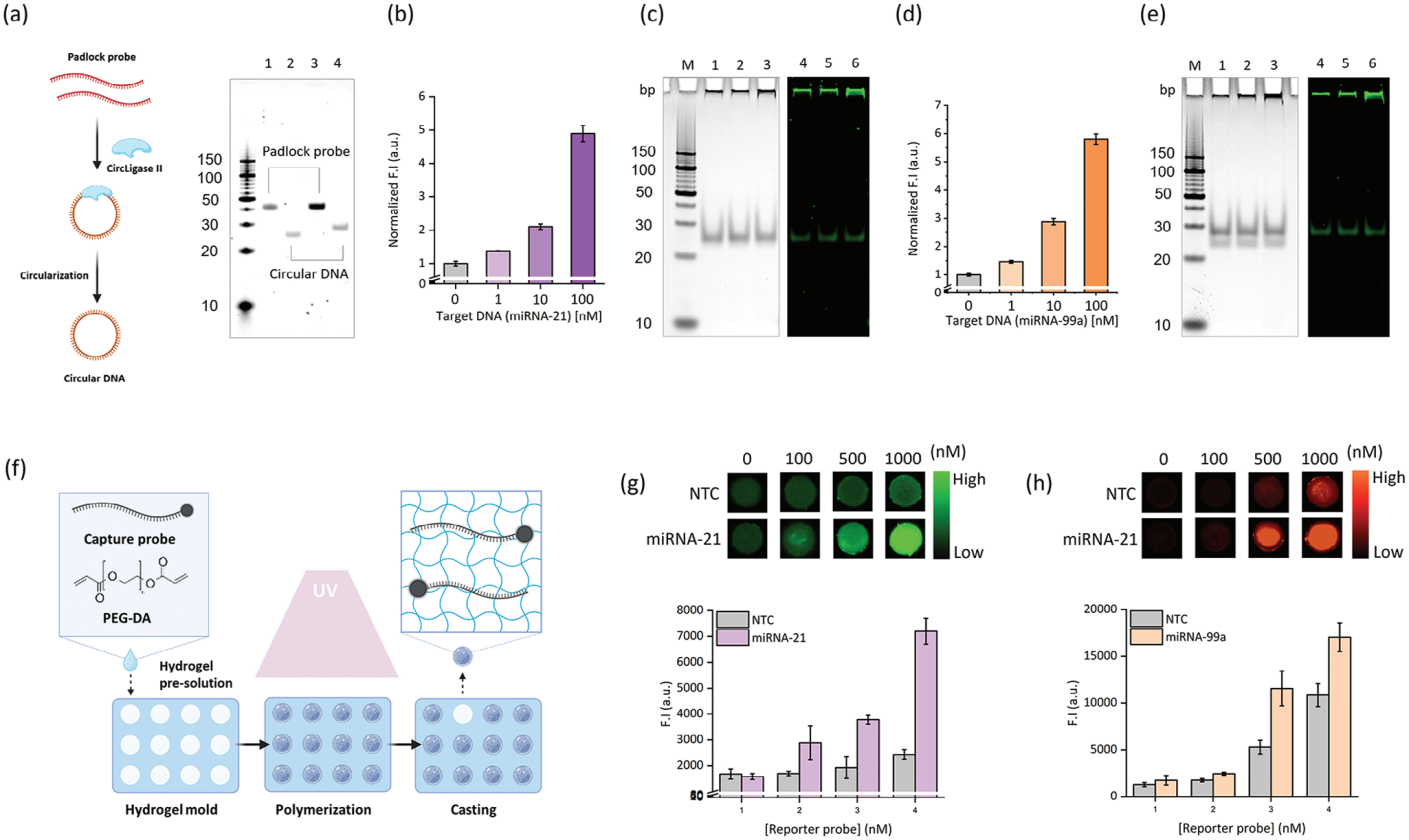
Mechanism of functioning of the nucleic acid amplification circuit. a) Schematic illustration of circular DNA synthesis. Polyacrylamide gel electrophoretic analysis of circular DNA and padlock probe. Lane 1 & 3: padlock probes individually targeting miRNA‐21 and miRNA‐99a; lane 2 & 4: circular DNA independently targeting miRNA‐21 and miRNA‐99a. b,d) Fluorescence intensity associated with the in‐solution nucleic acid amplification circuit containing various concentrations of (b) miRNA‐21 and (d) miRNA‐99a. c,e) Polyacrylamide gel electrophoresis analysis of the amplification products generated upon using different concentrations of (c) miRNA‐21 and (e) miRNA‐99a. Lane 1 & 4: 1 nm; lane & 2,5: 10 nm; lane 3 & 6: 100 nm; lane 1,2 & 3: GelRed staining; lane 4,5 & 6: SYBR Green II staining. f) Schematic illustration of the hydrogel casting process. g,h) Optimization of the NACH assay at different concentrations of the reporter probe (g: miRNA‐21; h: miRNA‐99a). Data represent mean ± standard deviation (SD) for three independent experiments. ***p < 0.0005, **p < 0.005.

In NACH, the RCA‐based nucleic acid amplification circuit induced exponential amplification of the target miRNAs by circular DNA. Circular DNA is formed through circularization of the padlock probe via the phosphodiester bond. **Figure** **2**a shows the mechanism of circular DNA synthesis using a padlock probe; the synthesized circular DNA was detected using native polyacrylamide gel (PAGE); 1 and 2 refer to the padlock probe and circular DNA of miRNA‐21; 3 and 4 refer to the padlock probe and circular DNA of miRNA‐99a. The reaction temperature, time, and enzyme concentrations critical to the isothermal amplification reaction were optimized (Figure S4, Supporting Information). Initially, dNTPs were used at concentrations ranging from 100 to 500 µm (Figure S4a, Supporting Information), and Phi29 DNA Polymerase was used at concentrations ranging from 2 to 12 units (Figure S4b, Supporting Information). These conditions were then applied to the NACH assay. Subsequently, the reaction was performed at temperatures ranging from 25 to 60 °C (Figure S4c, Supporting Information), and the reaction time was varied from 0 to 180 min (Figure S4d, Supporting Information). Based on these experimental results, the optimal conditions for the NACH assay were determined to be 400 µm for dNTP, 10 units for Phi29 DNA Polymerase, and 30 °C for the reaction temperature, 120 min for the reaction time. Before introducing it into the hydrogel, the performance of the RCA‐based nucleic acid amplification circuit was confirmed in the solution phase. When various concentrations of target miRNA‐21 and miRNA‐99a (0 to 100 nm) were used, the fluorescence intensity was found be positively related to the target miRNA concentration (Figure 2b,d). The measured fluorescence intensity (F.I.) was normalized by dividing it by the initial fluorescence intensity (F.I_0_) (Normalized F.I. = F.I/F.I_0_). In Figure 2c,e, the exponential amplification products were analyzed through native PAGE (1,2,3: GelRed staining; 4,5,6: SYBR Green II staining). The hybridization between the padlock probe, target miRNAs, and capture probe in the NACH was analyzed by PAGE (Figure S5, Supporting Information). Probes that exhibited binding affinity were used in the hydrogel for NACH. A schematic illustration of the process used to synthesize the capture probe and PEG‐DA‐based hydrogel is presented in Figure 2f. Fourier‐transform infrared spectroscopy revealed that PEG‐DA exhibited a strong absorption peak corresponding to the carbonyl group (C═O) of acrylate at 1732 cm^−1^ (comparator: polyethylene glycol (PEG)) (Figure S6, Supporting Information). To immobilize the capture probes within the hydrogel for the NACH assay, 100 nm capture probe was added to the hydrogel precursor solution containing PEG and PEG‐DA, and the mixture was then poured into a hydrogel mold and cured for 5 min with UV light irradiation (λ_ex_ = 365 nm). Subsequently, any remaining unbound residuals were removed by thoroughly rinsing with distilled water and then drying the sample. To confirm the immobilization of the capture probes within the hydrogel, capture probe anti‐sense were synthesized for the capture probes and reactivity tests were conducted. When fluorescently labeled capture probes anti‐sense were used within the hydrogel at concentrations ranging from 100 to 1000 nm, an increase in fluorescence intensity corresponding to the concentration in use was observed. Based on these results, it was confirmed that the capture probes targeting miRNA‐21 and miRNA‐99a were successfully incorporated within the hydrogel, as demonstrated by the corresponding capture probe anti‐sense independently labeled with FAM and Cy3 (Figure S7a,b, Supporting Information). The performance of the NACH assay with respect to target miRNA detection was evaluated using Chemidoc. Subsequently, the concentration of reporter probes within the hydrogel was optimized to ensure high sensitivity and specificity of the system. As shown in Figure 2g,h, the detection of miRNA‐21 and miRNA‐99a was optimized using various concentrations of reporter probes (0 to 1000 nm). Based on these initial experiments, reporter probes were finally used at concentrations of 1000 and 500 nm, respectively to detect miRNA‐21 and miRNA‐99a in the NACH. The NACH assay performed a washing step after the capture probe and reporter probe binding to remove unbound reporter probes, thereby reducing background signals.

### 2.3 Sensitivity and Specificity of the NACH Assay

**Figure 3:**
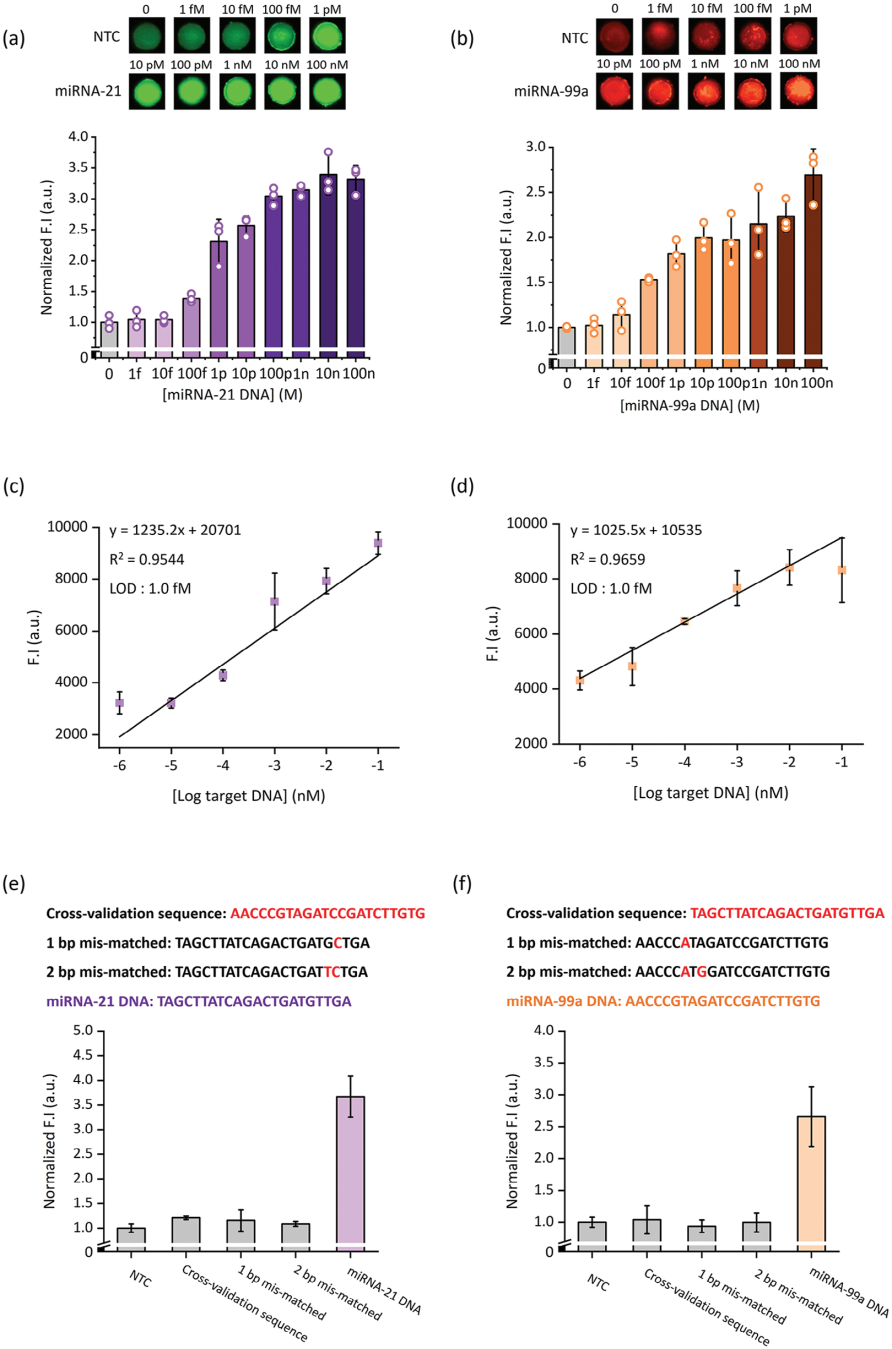
Detection of metastatic gastric cancer–associated biomarkers (miRNAs; miRNA‐21, and miRNA‐99a) using the NACH assay. a,b) The sensitivity of the NACH assay at various miRNA concentrations (1 fm to 100 nm) (a: miRNA‐21; b: miRNA‐99) (*n* = 3). c,d) Calibration curves corresponding to the estimated limit of detection (LOD) of the NACH assays for (c) miRNA‐21 and (d) miRNA‐99a. LOD (Formula: 3 × SD of linear regression (σ)/slope (m)). e,f) The selectivity and cross‐reactivity of the NACH assay for different target (cross‐validation sequence, 1 bp mis‐match, 2 bp mis‐match, and target DNA) (*n* = 3). Data represent mean ± standard deviation (SD) for three independent experiments.

We attempted to confirm the performance of the optimized NACH assay for detecting the target miRNAs. First, we evaluated the sensitivity of the NACH assay using various concentrations of target miRNAs (1 fm to 100 nm) and analyzed the fluorescence intensity of NACH using the ChemiDoc system (**Figure** **3**a,b). Results revealed that the fluorescence intensity of NACH increased as the target concentration increased. We estimated the fluorescence intensity to be linearly dependent on the log target miRNA concentration (Figure 3c,d). The linear fitted equations of miRNA‐21 and miRNA‐99a were, y = 1235.2x + 20 701 (R^2^ = 0.9544) and y = 1025.5x + 10 535 (R^2^ = 0.9659), respectively. Herein, y represents the fluorescence intensity and x represents the target concentration. Additionally, the following formula was used to calculate the limit of detection (LOD): LOD = 3 × sSD of linear regression/slope; the LOD of miRNA‐21 and miRNA‐99a was independently estimated to be 1 fm. Importantly, the NACH assay exhibited detection time and nucleic acid detection sensitivity comparable to those of previously studied isothermal amplification systems (Tables S2 and S3, Supporting Information). As shown in Figure 3e,f, genes with cross‐validation sequence and 1 bp mis‐matched and 2 bp mis‐matched were used as negative controls to evaluate specificity. High fluorescence intensity was confirmed in each assay for the target miRNA, indicating that this NACH assay exhibited high detection specificity. A similar trend was confirmed even when the test was repeated in triplicate (Figure S8, Supporting Information).

### 2.4 Performance of the NACH Assay in Pre‐Clinical Samples

**Figure 4:**
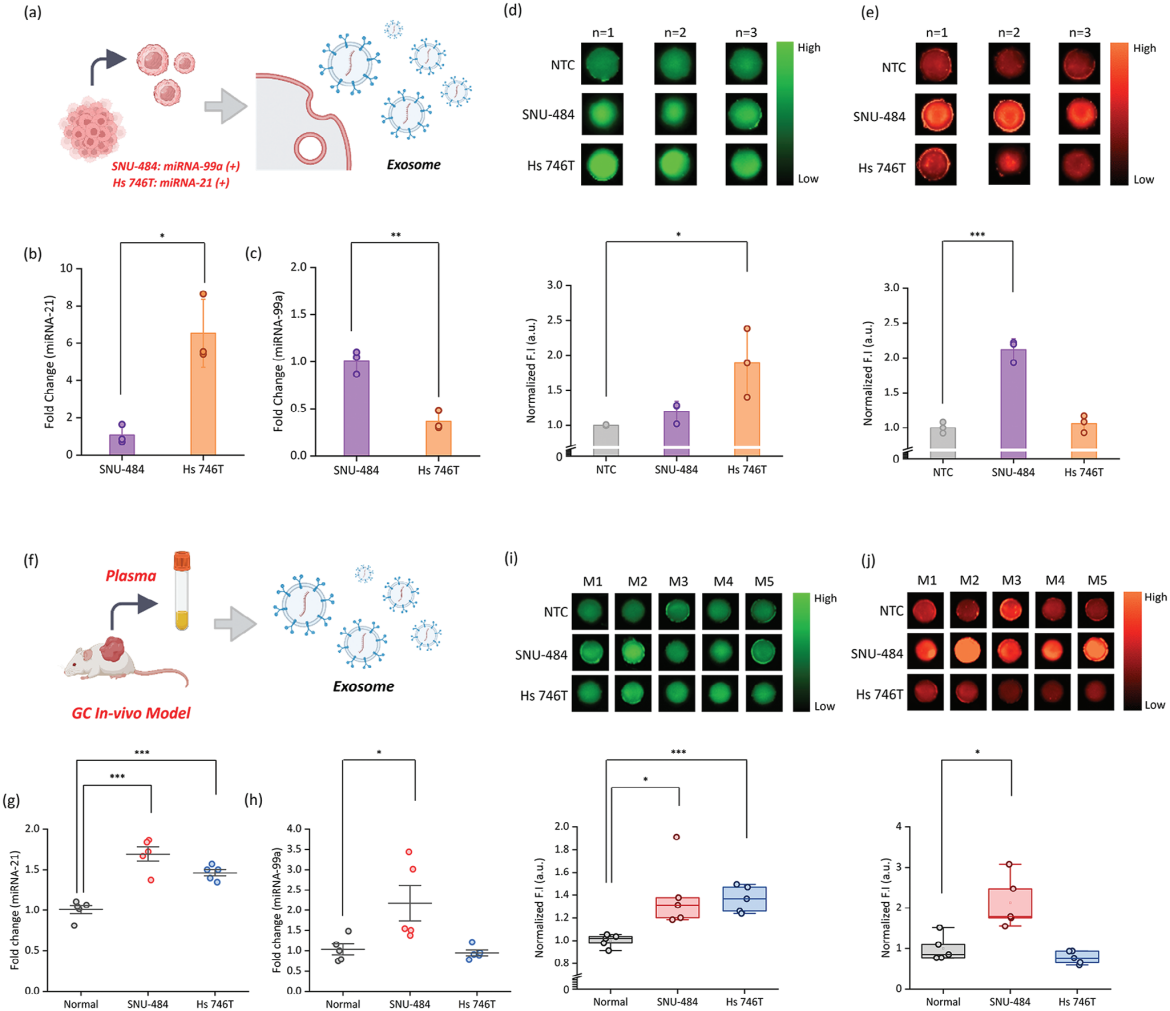
Performance of the NACH assay upon using pre‐clinical (in vitro and in vivo) models. a) Schematic diagram depicting the NACH assay for detecting exosomal miRNA in the culture media of gastric cancer cell lines. b,c) Quantitative real‐time PCR (qRT‐PCR) analysis of the expression of (b) miRNA‐21 and (c) miRNA‐99a in the culture media of gastric cancer cells. The expression levels of miRNA were normalized to those of miRNA‐16 and miRNA‐191 (reference genes). d,e) Fluorescence and associated intensity of (d) miRNA‐21 and (e) miRNA‐99a upon using total exosomal miRNA (80 ng per test) isolated from the culture media of gastric cancer cells (*n* = 3). Total exosomal miRNA was isolated from the plasma of three different mouse groups (Normal, SNU‐484, and Hs 746T). f) Schematic diagram depicting the strategy used for the detection of target miRNA in exosomes isolated from the plasma of mice transplanted with gastric cancer cell lines using the NACH assay. g,h) qRT‐PCR analysis of target miRNA expression (g: miRNA‐21; h: miRNA‐99a) in exosomes extracted from the plasma of mice transplanted with gastric cancer cell lines. i,j) Fluorescence and associated intensity of (i) miRNA‐21 and (j) miRNA‐99a upon using total exosomal miRNA (5 µL/hydrogel) from the plasma of mice transplanted with gastric cancer cell lines (*n* = 5). Data represent mean ± SD for independent experiments. ***p < 0.0005, **p < 0.005, *p < 0.05.

EMT is an important factor in the progression, resistance, and recurrence of cancer.^[^ ^17^ ^]^ Notably, studies have shown that EMT is closely associated with the expression levels of miRNAs.^[^ ^18^ ^]^ We have identified miRNA‐99a as a diagnostic biomarker closely related to EMT in GC, and we evaluated the changes in the expression levels of miRNA‐21 (as an oncogene) in cancer. We analyzed the differences in the expression of potential reference genes to evaluate the performance of NACH in pre‐clinical samples, i.e., SNU‐484 (Figure S9a, Supporting Information) and Hs 746T (Figure S9b, Supporting Information) cell lines. Our results confirmed that genes encoding miRNA‐16 and miRNA‐191 are consistently expressed regardless of the cell line. Based on these results, we used the genes encoding miRNA‐16 and miRNA‐191 as reference genes in qRT‐PCR for both pre‐clinical and clinical samples. Distinctive expression signatures of these miRNAs (miRNA‐21 and miRNA‐99a) have been identified in different GC cells (Figure S10, Supporting Information). It was confirmed that miRNA‐21 and miRNA‐99a are upregulated in Hs 746T and SNU‐484 cells, respectively (Figure S10a,b, Supporting Information). Among the various GC cell lines, miRNA‐99a exhibited relatively higher expression in the SNU‐484 and Hs 746T cells. Based on this observation, we selected SNU‐484 and Hs 746T as models of GC cells to evaluate their pre‐clinical performance in the NACH assay. Cellular RNA was extracted from cells and used in the NACH assay to detect the target miRNAs by measuring the increase in fluorescence intensity (Figure S11, Supporting Information). We also analyzed the expression of miRNA‐21 and miRNA‐99a in cells using qRT‐PCR (Figure S11a,b, Supporting Information). Negligible fluorescence was observed in the non‐target control (NTC; buffer without RNA) and individual control samples. However, strong fluorescence (and corresponding intensity) were observed for miRNA‐21‐specific NACH in Hs 746T cells and miRNA‐99a‐specific NACH in SNU‐484 cells (Figure S11c,d, Supporting Information), thereby confirming an expression pattern similar to that observed in the qRT‐PCR. Furthermore, the expression of exosomal miRNAs extracted from each culture medium exhibited a pattern similar to that observed for the cellular RNA (**Figure** **4**a). Comparison of miRNA expression levels between the two cell lines using qRT‐PCR revealed a 6.0‐fold higher expression of miRNA‐21 in the Hs 746T cell line and a 3.0‐fold higher expression of miRNA‐99a in the SNU‐484 cell line (Figure 4b,c). Fluorescence intensity of these exosomal miRNAs was analyzed using the NACH assay. When exosomal miRNA from cell lines over‐expressing each target miRNA was treated, stronger fluorescence was observed compared to the control group, including NTC, and a significant increase in analyzed fluorescence intensity was also confirmed (Figure 4d,e). This trend was consistent with the trends observed in the qRT‐PCR, confirming the NACH assay's reliability. Next, using the confirmed performance, blood samples (plasma) from in vivo models were employed in the NACH assay. As mentioned above, we established two types of GC models in mice by independently transplanting SNU‐484 and Hs 746T cells.

To initially assess the expression of target miRNAs within the tumor tissue, tumors were excised from in vivo models. RNA extracted through homogenization was then subjected to qRT‐PCR and the NACH assay to detect individual miRNAs (Figure S12, Supporting Information). As expected, the results observed in both analyses were correlated with the in vitro results. In qRT‐PCR, miRNA‐21 was found to be overexpressed by 25.0‐fold in the tumor of the Hs 746T group, while miRNA‐99a was ≈1.6‐fold overexpressed in the tumor of the SNU‐484 group (Figure S12a,b, Supporting Information). The NACH assay revealed similar trends, with each target miRNA detected with strong fluorescence and higher intensity (Figure S12c,d, Supporting Information). Additionally, plasma was obtained from in vivo models, and the exosomal miRNA extracted from this plasma was used for the NACH assay (Figure 4f), while plasma from healthy mice (normal) was used as a control. qRT‐PCR was also performed on this RNA. miRNA‐21 exhibited a 1.7‐fold and 1.5‐fold upregulation in the plasma from both mouse models (compared to the normal). Furthermore, plasma obtained from the SNU‐484 group showed higher miRNA‐21 expression than plasma obtained from the Hs 746T group (Figure 4g). miRNA‐99a was upregulated 2.0‐fold in the SNU‐484 group (compared to normal and Hs 746T group), confirming a pattern similar to that observed in vitro (Figure 4h). This trend was also confirmed when in our NACH assay. In both groups, higher fluorescence and fluorescence intensities were observed in the NACH assay for miRNA‐21 detection (compared to normal) (Figure 4i). In the NACH assay for miRNA‐99a detection, higher fluorescence was observed in samples from the SNU 484 group compared to that observed in samples from the Hs 746T and the normal groups (Figure 4j).

To assess the clinical applicability of the NACH assay and compare its performance with qRT‐PCR, it is essential to evaluate target miRNA (miRNA‐21 and miRNA‐99a) detection. Therefore, despite differences in the expression patterns of the target miRNAs between tumors extracted from in vivo models and those extracted from blood (plasma), we opted to proceed with performance evaluation using clinical samples, considering the similar detection tendencies of the qRT‐PCR and the NACH assay.

### 2.5 Performance of the NACH Assay Using GC Patient Samples

**Figure 5:**
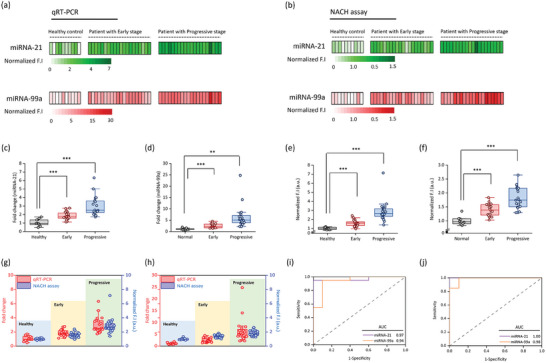
The performance of the NACH assay upon using clinical samples. Heatmap of miRNA expression levels in plasma samples obtained from healthy individuals and patients with early‐stage and progressive‐stage gastric cancer (GC) using a) qRT‐PCR and b) NACH assay. In the heatmap, fluorescence intensities represent the amount of miRNA‐21 (green) and miRNA‐99a (red) present in the exosomal miRNA. c,d) Quantitative real‐time PCR (qRT‐PCR) analysis of (c) miRNA‐21 and (d) miRNA‐99a expression in the plasma of healthy individuals and patients with early‐stage and progressive‐stage GC (*n* = 20). The expression levels of miRNA were normalized to those of miRNA‐16 and miRNA‐191. e,f) Fluorescence and associated intensity of (e) miRNA‐21 and (f) miRNA‐99a upon using total exosomal miRNA (5 µL per test) isolated from the plasma of healthy individuals and patients with early‐stage and progressive‐stage GC. (*n* = 20). g,h) Comparison of the performance of the qRT‐PCR and NACH assays for detecting (g) miRNA‐21 and (h) miRNA‐99a in various stages of GC. i,j) Area under the curve values for miRNA‐21 and miRNA‐99a in (i) early‐stage and (j) progressive‐stage GC. ***p < 0.0005, **p < 0.005.

We obtained blood (plasma) samples from GC patients at both early and advanced stages of GC (Early: 20; Progressive: 20); plasma samples were also obtained from healthy individuals (Healthy: 10). Information regarding the healthy individuals and patients is shown in Table S4 (Supporting Information). Exosomal miRNA (5 µL) extracted from clinical samples were used in the NACH assay. We have validated the reliability of the results obtained using the NACH assay and qRT‐PCR and presented the same as a heatmap for intuitive confirmation. **Figure** **5**a presents a heatmap depicting fold‐change values for miRNA‐21 and miRNA‐99a in qRT‐PCR. Figure 5b presents a heatmap depicting normalized fluorescence intensity corresponding to miRNA‐21 and miRNA‐99a in the NACH assay. The data obtained from each analysis allow for clear comparison and visualization. We confirmed that both miRNA‐21 and miRNA‐99a were upregulated in the GC patient groups (compared to healthy individuals). This upregulation was more pronounced with the progression of cancer, as determined by qRT‐PCR (Figure 5c,d). Particularly, miRNA‐21 exhibited ≈2.0‐fold higher expression in the early stage and 3.0‐fold higher in the progressive stage. Additionally, miRNA‐99a exhibited an ≈3.0‐fold upregulation in the early stage and 5.0‐fold upregulation in the progressive stage. A similar pattern was observed in the NACH assay (Figure 5e,f); the hydrogel images of the clinical samples are presented in Figure S13 (Supporting Information). Notably, a direct comparison of the performance of qRT‐PCR and the NACH assay with respect to the fold‐change and normalized fluorescence intensity of miRNA‐21 and miRNA‐99a in clinical samples yielded consistent results (Figure 5g,h, respectively). We observed similar increase in expression levels in both early and progressive stages (compared to the healthy group). Particularly, the receiver operating characteristic curve for determining the performance of these assays at detecting miRNA‐21 and miRNA‐99a at each stage revealed high diagnostic accuracy. The area under the curve (AUC) value was 0.97 for miRNA‐21 and 0.94 for miRNA‐99a in the early stage. In the progressive stage, the AUC value was 1.00 for miRNA‐21 and 0.98 for miRNA‐99a (Figure 5i,j). Also, based on the analysis of clinical samples, we analyzed the statistical data for qRT‐PCR and the NACH assay. The True Positive Rate (TPR) for qRT‐PCR was analyzed as 85% in the early stage and 100% in the progressive stage, while the TPR for the NACH assay was analyzed as 95% and 100%, respectively. Additionally, the True Negative Rate (TNR), representing specificity, was consistently analyzed as 90% in the normal group (Figure S14, Supporting Information). These cut‐off values accurately differentiate between miRNA‐21 and miRNA‐99a with high sensitivity and specificity in the NACH assay. Consistent results were observed across GC patients, demonstrating a strong correlation with the results obtained using qRT‐PCR. These results suggest the potential of the NACH assay as a valuable clinical tool for miRNA‐based GC diagnosis.

### 2.6 NACH Assay with a Portable Fluorometer

**Figure 6:**
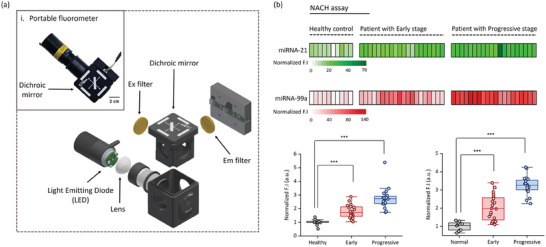
Assessment of the use of clinical samples in the nucleic acid amplification circuit‐based hydrogel (NACH) assay by employing a portable fluorometer. a‐i) Photograph of the portable fluorometer, a‐ii) Schematic depicting the internal structure of the portable fluorometer. b) Fluorescence intensity corresponding to miRNA‐21 (green) and miRNA‐99a (red) in samples derived from healthy individuals (*n* = 10) and patients with early‐stage (*n* = 20) and progressive‐stage (*n* = 20) gastric cancer, evaluated using the NACH assay along with a portable fluorometer. Fluorescence intensity represents the amount of miRNA‐21 (green) and miRNA‐99a (red) present in the total exosomal miRNA obtained from GC patient samples. ***p < 0.0005.

Despite numerous studies focusing on utilizing cancer biomarkers for point‐of‐care detection,^[^ ^19^ ^]^ implementing these applications on a point‐of‐care testing (POCT) platform using clinical samples remains challenging. Here, the hydrogel exhibits controllability and flexibility through photo‐polymerization, enabling its use in designing fluorescent sensors that emit fluorescence within the hydrogel in a one‐step reaction. Therefore, we developed a portable fluorometer capable of measuring the fluorescence of the hydrogel, as depicted in Figure S15 (Supporting Information). This device is designed to detect specific wavelengths. Equipped with a handle for easy mounting, this portable fluorometer contains emission (λ_em_) and excitation (λ_ex_) filters positioned on either side. Consequently, the device emits light through a dichroic mirror, enabling the observation of fluorescence at specific wavelengths (**Figure** **6**a). A photograph of the portable fluorometer is presented in Figure 6a‐i, while the internal structure of the portable fluorometer is schematically presented in Figure 6a‐ii. NACH assay was conducted to detect individual miRNAs in pre‐clinical (in vivo) and clinical samples using a portable fluorometer that enables the acquisition of fluorescent images. These images were then analyzed using Image J to detect miRNA‐21 and miRNA‐99a based on the intensity of green (FAM) and red (Cy) color, respectively. The analyzed fluorescence intensities were represented in a heatmap (Figure 6b; Figure S16, Supporting Information). NACH assay using the portable fluorometer revealed that the expression trends of miRNA‐21 and miRNA‐99a were similar to those observed upon using qRT‐PCR and fluorescence imaging equipment. This suggests that the NACH assay with a portable fluorometer can potentially serve as a diagnostic tool for use in POCT.

## 3 Conclusion

We propose a simple yet powerful tool for detecting exosomal miRNA derived from metastatic GC, i.e., the NACH assay. The NACH assay is designed to detect fluorescent signals through DNA amplification reactions within a hydrogel. This assay integrates probe binding, amplification, and detection via fluorescent signals into a one‐step reaction. Notably, the amplification reaction facilitated by DNA molecule binding within the hydrogel enables the detection of target miRNA with high sensitivity. These advantages of the NACH assay allow for the rapid, simple, and accurate detection of target miRNA. The assay demonstrated excellent performance, achieving a LOD of 1.0 fm for miRNA‐21 and miRNA‐99a. We observed that the NACH assay could detect exosomal miRNA in culture media and mouse plasma with high sensitivity (compared to qRT‐PCR). Upon using human plasma, the NACH assay exhibited high GC diagnostic accuracy, with AUCs of 0.97 and 1.00 for miRNA‐21 and 0.94 and 0.98 for miRNA‐99a in the early and progressive stages, respectively. Notably, we analyzed the performance of the NACH assay along with a portable fluorometer for detecting target miRNAs in mouse and human plasma samples. This assay does not require expensive equipment, extensive handling time, or expertise. However, performing quantitative analysis using the RCA strategy employed in the NACH assay is difficult. Therefore, future research would focus on clinically validating stable fluorescence intensity and reproducibility in more plasma samples for precise analysis. Overall, the NACH assay is a promising tool for high‐sensitivity diagnosis of early‐ and advanced‐stage cancers, offering high specificity and simple detection. The use of a portable fluorometer also enables the potential employments of this strategy in POCT, enabling sensitive and specific diagnostics in the field. These advantages suggest that the NACH assay could be further engineered and customized to detect the expression of multiple genes in metastatic cancer.

## 4 Experimental Section

### Materials

Poly(ethylene glycerol) (Mn 3350; PEG 3.35 K), n,n‐diisopropylethylamine (DIPEA), dichloromethane (DCM), and the radical photo‐initiator 2‐hydroxy‐2‐methylpropiophenone (HMPP) were obtained from Sigma‐Aldrich (USA). Acryloyl chloride was procured from Tokyo Chemical Industry Co. All oligonucleotides were purchased from Bioneer Co. CircLigase II ssDNA Ligase (100 U µL^−1^) was purchased from Biosearch Technologies. Phi29 DNA Polymerase (10 U µL^−1^) and 10 × Phi29 DNA Polymerase buffer were purchased from New England Biolabs. dNTPs (2.5 mm) and SYBR Green II were purchased from TaKaRa. Tween 20 and sodium chloride were purchased from Sigma‐Aldrich. GelRed (10000×) was purchased from Biotium Co. TEMED, 10% APS, and 40% acrylamide/bis solution (29:1) were purchased from Bio‐Rad Co. TBE buffer (10×) was purchased from Biosesang. UltraPure distilled water was purchased from Invitrogen. Furthermore, RPMI‐1640 medium, Dulbecco′s Modified Eagle′s Medium (DMEM), fetal bovine serum (FBS) were purchased from Thermo Fisher Scientific Inc (USA). miRNeasy Mini Kit, exoRNeasy Midi Kit, miCURY LNA reverse transcription (RT) Kit and miRCURY LNA SYBR Green PCR Kit were purchased from Qiagen.

### PEG‐DA Synthesis

The hydrogel backbone was synthesized using PEG‐DA by referring to a method reported in published studies.^[^ ^20^ ^]^ First, 60 g PEG (molecular weight: 3,350 Da) was completely dissolved in 75 mL DCM. When the solution became transparent, 7 mL DIPEA was added. Then, 6.5 mL acryloyl chloride was gradually added to mixture in a dropwise manner while stirring on ice. The mixture was stirred overnight in the dark at 4 °C under vacuum. Subsequently, the mixture was precipitated and filtered in 1 L diethyl ether, and the filtrate was subjected to vacuum drying to obtain a powder. This powder was dissolved in 75 mL DCM and 500 mL potassium carbonate (2 M). The solution was left overnight for phase separation to remove the by‐products. The lower layer was filtered, precipitated in 1 L diethyl ether, and dried under vacuum. The synthesized PEG‐DA was stored at 4 °C for further use.

### Hydrogel Fabrication Using Capture Probe

First, cylindrical hydrogels were developed through the photo‐polymerization of PEG‐DA. The master template with vertical column patterns was fabricated using 3D printing, and the hydrogel mold was constructed via soft lithography using the master template and PDMS. The PDMS mixture containing PDMS base and curing agent in a 10:1 weight ratio was poured onto the master template and heated at 80 °C for 6 h. Subsequently, 20 µL hydrogel precursor solution (20% [w/v] PEG‐DA, 20% [w/v] PEG, 60% [v/v] 1× TE buffer, and 0.1% [v/v] HMPP) was poured onto the hydrogel mold, and photo‐polymerization was carried out by UV irradiation (254 nm) 5 min. The resulting cylindrical hydrogels were rinsed in distilled water for 1 h and stored at 4 °C for further use. To generate hydrogels incorporating capture probes, acrylamide was synthesized at the ends of the capture probes. Subsequently, the capture probes were mixed with the hydrogel precursor solution, and 20 µL of the mixture was poured into the hydrogel mold, ultimately producing hydrogels incorporating capture probes. Afterward, the unbound capture probes were removed by rinsing with distilled water for 1 h, and finally, a hydrogel with a capture probe was obtained.

### NACH Assay

The NACH assay employed rolling circle‐mediated isothermal amplification. The sequences utilized in the current system are listed in Table S1 (Supporting Information). The total reaction volume (20 µL) comprised circular DNA (5 µL), Phi29 DNA Polymerase (1 µL), 10× Phi29 DNA Polymerase buffer (2 µL), dNTPs (4 µL), reporter probes (2 µL), various concentrations of the target (In vitro, In vivo, and clinical samples), and nuclease‐free water. This sample solution was first mixed and then incubated with the fabricated hydrogel at 30 °C for 2 h. Subsequently, unbound reporter probes were removed by washing with TET buffer containing 0.05% Tween 20 and 50 mm NaCl at room temperature for 30 min. Finally, the fluorescence emitted from the NACH was acquired and analyzed using ChemidocTM (Bio‐Rad). Fluorescence intensity corresponding to miRNA‐21 (λ_ex_ = 495 nm, λ_em_ = 520 nm) and miRNA‐99a (λ_ex_ = 550 nm, λ_em_ = 570 nm) was measured, and the increase in fluorescence intensity was calculated by normalization (F.I/F.I_0_).

### Cell Culture

SNU‐484 (KCLB No.00484) and Hs 746T (KCLB No.30135) cells were obtained from the Korean Cell Line Bank (Republic of Korea) and cultured using recommended methods. SNU‐484 cells were cultured in RPMI‐1640 medium, while Hs 746T cells were cultured in Dulbecco's modified Eagle's medium. Both cell lines were maintained in a humidified incubator at 37 °C in an atmosphere of 5% CO_2_. All complete culture media were supplemented with 10% [v/v] fetal bovine serum.

### In Vivo Models of GC

All animal experiments were conducted with the approval of the Institutional Animal Care and Use Committee of the Yonsei Laboratory Animal Center (IACUC 2019‐0317). Six‐week‐old male nude BALB/c mice (*n* = 5; average weight 22 ± 2 g) were purchased from Orient (Korea). SNU‐484 and Hs 746T cells (1 × 10^7^ cells) resuspended in 60 µL serum‐free media were independently injected into the proximal thigh region of male nude BALB/c mice using a 29 G insulin syringe. The mice were observed until the tumor volume reached 1000 mm^3^ or was ulcerated. Tumor size was assessed three times per week and calculated using the formula [4/3) × π × (minor axis/2)^2^ × (major axis/2) mm^3^]. Untreated healthy male nude BALB/c mice (*n* = 5) were used as a control. Plasma and tissue samples were collected from these mice for subsequent analyses.

### miRNA Extraction

Cellular miRNA was extracted using the miRNeasy kit (Qiagen) according to the manufacturer's instructions. Mouse tumors were lysed in QIAzol lysis reagent using a tissue grinder, and total RNA was extracted using the miRNeasy kit (Qiagen) according to the manufacturer's instructions. Exosomal miRNAs isolated from 100 µL human and mouse plasma were extracted using the exoRNeasy Midi kit (Qiagen) and purified according to the manufacturer's protocol. Subsequently, the extracted RNA was quantified using Nanodrop OneTM (Thermo Fisher Scientific).

### qRT‐PCR

The miRNAs purified above were reverse transcribed into cDNA using the miRCURY LNA RT Kit (Qiagen). The RT reaction (10 µL) comprised 5× miRCURY RT reaction buffer (2 µL), 10× miRCURY RT enzyme mix (2 µL), miRNA (1 µL), and nuclease‐free water (5 µL). The cycling conditions for RT included 42 °C for 60 min followed by inactivation at 95 °C for 5 min. The synthesized cDNA was stored at 4 °C for further use. The qRT‐PCR system comprised 2× miRCURY SYBR Green master mix (5 µL), PCR primer mix (1 µL), ROX reference dye (0.05 µL), diluted cDNA template (2 µL), and nuclease‐free water (1.95 µL). The reactions were run on a CFX96TM Real‐Time PCR System (Bio‐Rad). The following cycling conditions were used: 95 °C for 2 min, followed by 45 cycles of 95 °C for 10 s and 56 °C for 60 s. The expression of target miRNA was normalized to that of miRNA‐16 and miRNA‐191.

### PAGE

PAGE samples were run on 10% native polyacrylamide gels with the following conditions: buffer, TBE; voltage, 60 V; time, 120 min. Subsequently, the gels were stained with GelRed for 30 min. Images were captured using Chemidoc^TM^ (Bio‐Rad).

### Clinical Sample Acquisition

Human plasma samples (*n* = 50) collected from GC patients were provided by the Biobank of Ajou University Hospital (approval No. AJHB‐2023‐03) and the study protocol was approved by the Ethics Committee of the Korea Research Institute of Bioscience and Biotechnology (IRB #2022‐1317‐077). The study included 40 patients with a clinical diagnosis of GC and 10 healthy individuals. Patient samples were classified into early stage and progressive stage based on the pathological results obtained upon using endoscopic mucosal resection. According to the ICD‐10 guidelines, GC patients were medically classified as C1691 and C1690, while healthy individuals were classified as Z00.

### Development of a Portable Fluorometer for POCT

A portable fluorometer was developed for FAM (λ_ex_: 495 nm; λ_em_: 520 nm) and Cy3 (λ_ex_: 550 nm; λ_em_: 570 nm) to analyze the fluorophore intensity within the hydrogel. For the portable fluorometer used to detect FAM, a blue light emitting diode (LED) (490 nm) filtered using a 483 nm bandpass filter with a 31 nm bandwidth and an optical density (OD) of 6 was used as the excitation source. A dichroic filter with a cutoff wavelength of 506 nm was used to reflect the excitation beam and transmit only the emission beam; this resulted in the delivery of the excitation beam into the sample and transmission of the fluorescent light from the sample to the photodetector. A bandpass filter of 565 nm filtered the emission wavelength with a 43 nm bandwidth and an OD of 6. For the portable fluorometer used to detect Cy3, a 530 nm green LED light source and a 535 nm bandpass filter with a 43 nm bandwidth and an OD of 6 were used for the excitation beam. A dichroic filter with a cutoff wavelength of 562 nm and a bandpass filter with a center wavelength of 592 nm, a bandwidth of 43 nm, and an OD of 6 were used to obtain the emission beam. All images were acquired using a CMOS QSXGA USB camera.956142

### Statistical Analysis

All experiments were independently conducted at least thrice; the number of replicates was mentioned in each graph. Data were reported as mean ± SD. The following legend was added to each figure: ***p < 0.0005, **p < 0.005, *p < 0.05. LOD was calculated using the following equation: LOD = 3σ/m, where σ is the SD of the blank sample, and m is the slope of the data fitted in a linear range. The terms FPR (False Positive Rate), FNR (False Negative Rate), TNR (True Negative Rate), TPR (True Positive Rate), PPV (Positive Predictive Value), and NPV (Negative Predictive Value) were defined as follows. Each value was calculated using the following formulas: FPR = FP/(TP + FP), FNR = FN/(TP + FN), TNR = TN/(TN + FP), TPR = TP/(TP + FN), PPV = TP/(TP + FP), NPV = TN/(TN + FN). Sensitivity and specificity correspond to TPR and TNR, respectively, while PPV and NPV represent the positive and negative predictive values.

## Conflict of Interest

The authors declare no conflict of interest.

## Supporting information



Supporting Information

## Data Availability

The data that support the findings of this study are available from the corresponding author upon reasonable request.
