# Supplementary material for: Nucleic Acid Amplification Circuit‐Based Hydrogel (NACH) Assay for One‐Step Detection of Metastatic Gastric Cancer‐Derived Exosomal miRNA
Source: Adv Sci (Weinh). 2024 Sep 23;11(43):2407621. doi: 10.1002/advs.202407621 (PMC11578299; doi:10.1002/advs.202407621)
Supplement: Supplementary file 1 — Supporting Information [file ADVS-11-2407621-s001.docx]

Supporting Information

**Nucleic Acid Amplification Circuit-Based Hydrogel (NACH) Assay for One-step Detection of Metastatic Gastric Cancer-Derived Exosomal miRNA**

*Seung Beom Seo, Jaewoo Lim, Kyujung Kim, Inhee Maeng, Hyun Wook Rho, Hye Young Son, Eunjung Kim, Eunji Jang, Taejoon Kang, Juyeon Jung, Seung Jae Oh*, Yong-Min Huh* and Eun-Kyung Lim**


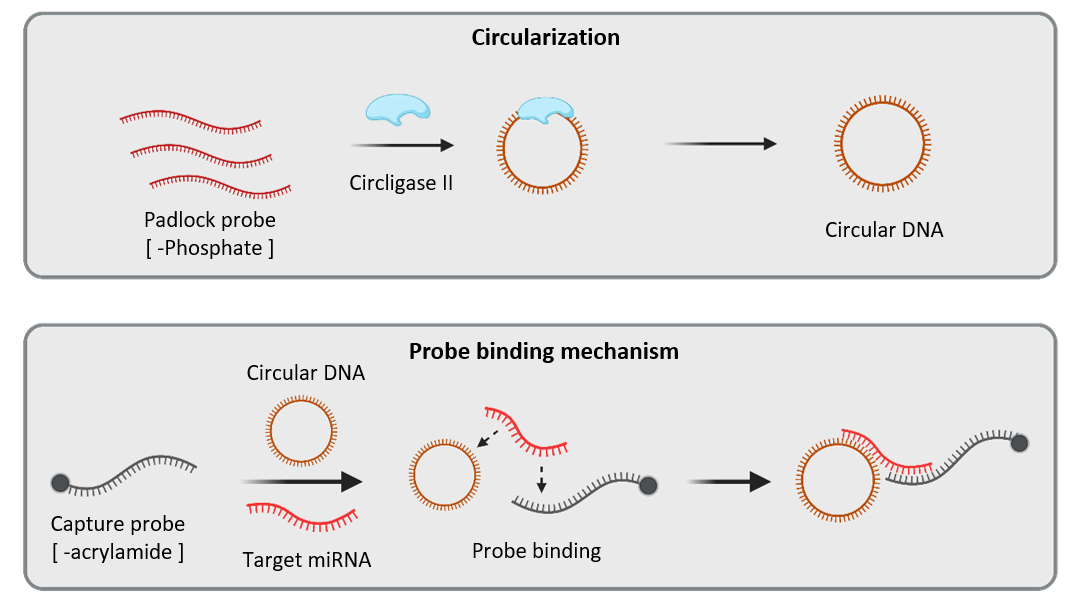


**Figure S1**. Probe binding mechanism for the nucleic acid amplification circuit.

| **Probe pair** | **miRNA-21** | **miRNA-99a** |
| --- | --- | --- |
| **Padlock probe** | 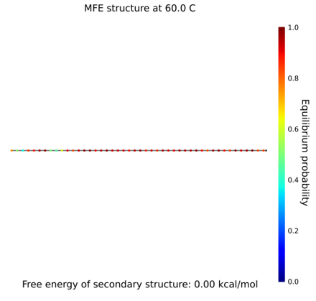 | 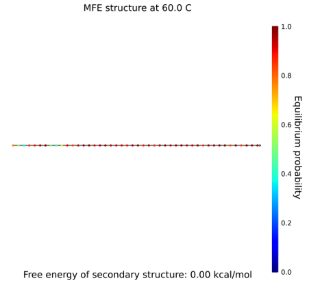 |
| **Capture probe + Target DNA** | 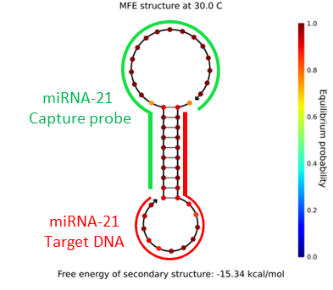 | 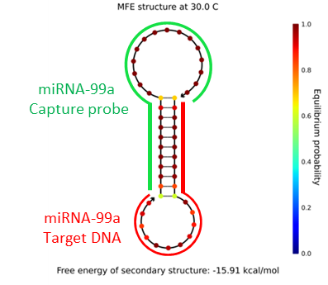 |
| **Capture probe + Target DNA + Padlock probe** | 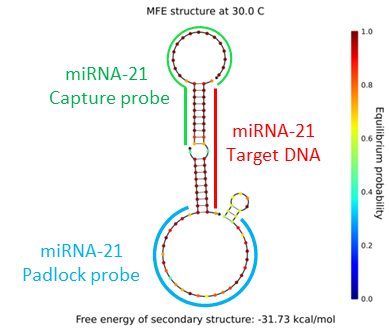 | 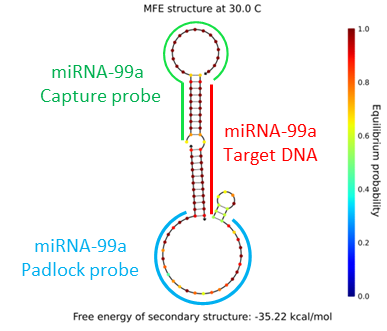 |

**Figure S2**. Alignment and thermodynamic properties of the amplification probes in the NACH assay analyzed using the Nucleic Acid Package (NUPACK). No secondary structures were observed in case of the padlock probes for miRNA-21 and miRNA-99a at 60 °C. Confirmation that the target miRNA mediates the binding of the capture probe and the padlock probe at 37 °C. The concentration analyzed in NUPACK is the same under experimental conditions.

| miRNA-21 (Total 22 bp) | miRNA-99a (Total 22 bp) |
| --- | --- |
| Capture probe (11 bp) - Target (22 bp) - Circular DNA (11 bp) | Capture probe (11 bp) - Target (22 bp) - Circular DNA (11 bp) |
|  |  |
| Capture probe (5 bp) - Target (22 bp) - Circular DNA (17 bp) | Capture probe (5 bp) - Target (22 bp) - Circular DNA (17 bp) |
|  |  |
| Capture probe (17 bp) - Target (22 bp) - Circular DNA (5 bp) | Capture probe (17 bp) - Target (22 bp) - Circular DNA (5 bp) |
|  |  |

**Figure S3.** Comparison of sequence and structure design according to sequence length using Nupack software.


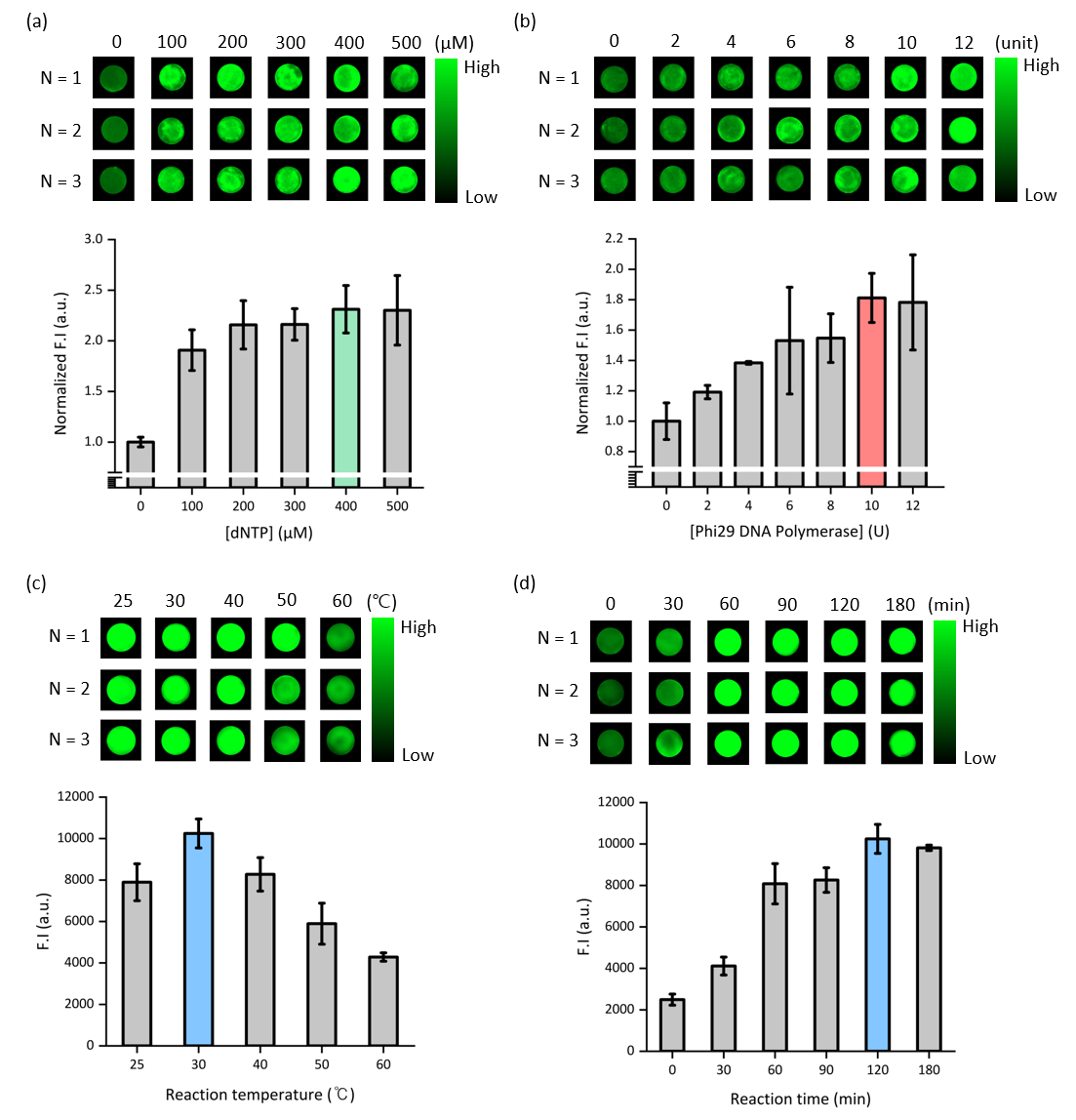


**Figure S4.** Optimization of the NACH assay. (a) Optimization through fluorescence intensity for various concentrations of dNTPs. (b) Optimization through fluorescence intensity for various concentrations of Phi29 DNA Polymerase. (c) Optimization through fluorescence intensity of NACH reaction temperature (℃). (d) Optimization through fluorescence intensity of NACH reaction time (min).


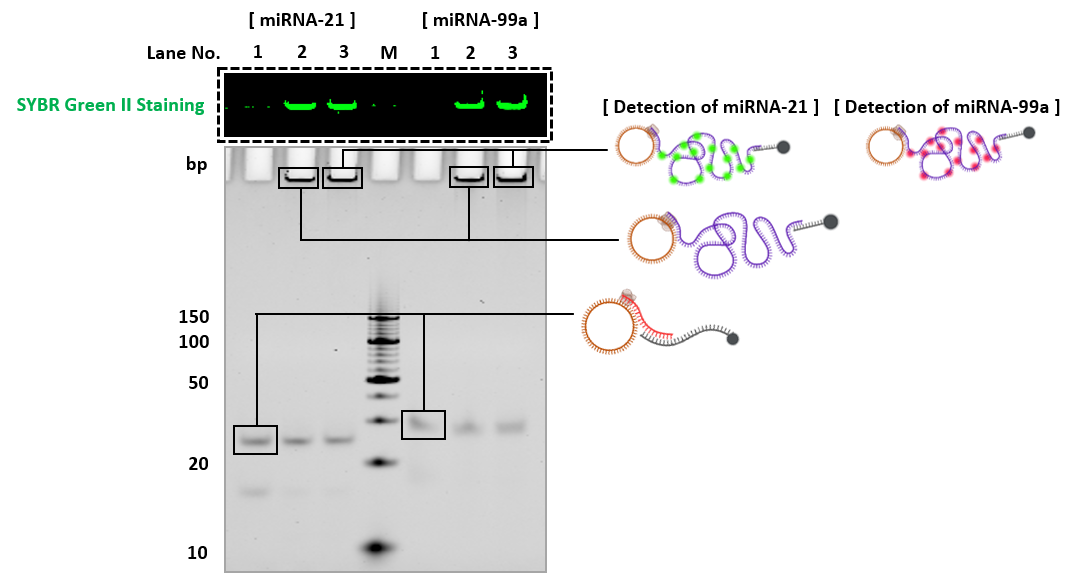


**Figure S5**. Polyacrylamide gel electrophoresis-based analysis of the probe binding in the NACH assay. 1: Binding between circular DNA and capture probe mediated by the target miRNAs (miR-21 and miR-99a); 2: Amplification products of miR-21 and miR-99a in the NACH assay, 3: Amplification products with reporter probe hybridized in the NACH assay.


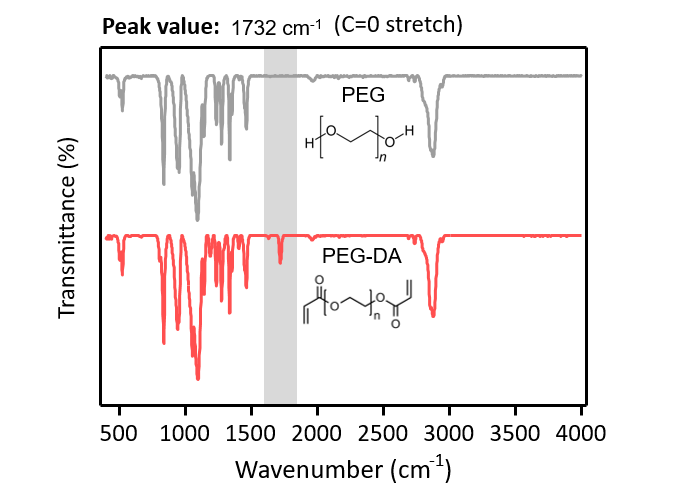


**Figure S6**. Fourier Transform Infrared Spectroscopy (FTIR) spectra of PEG (gray) and PEG-DA (red).


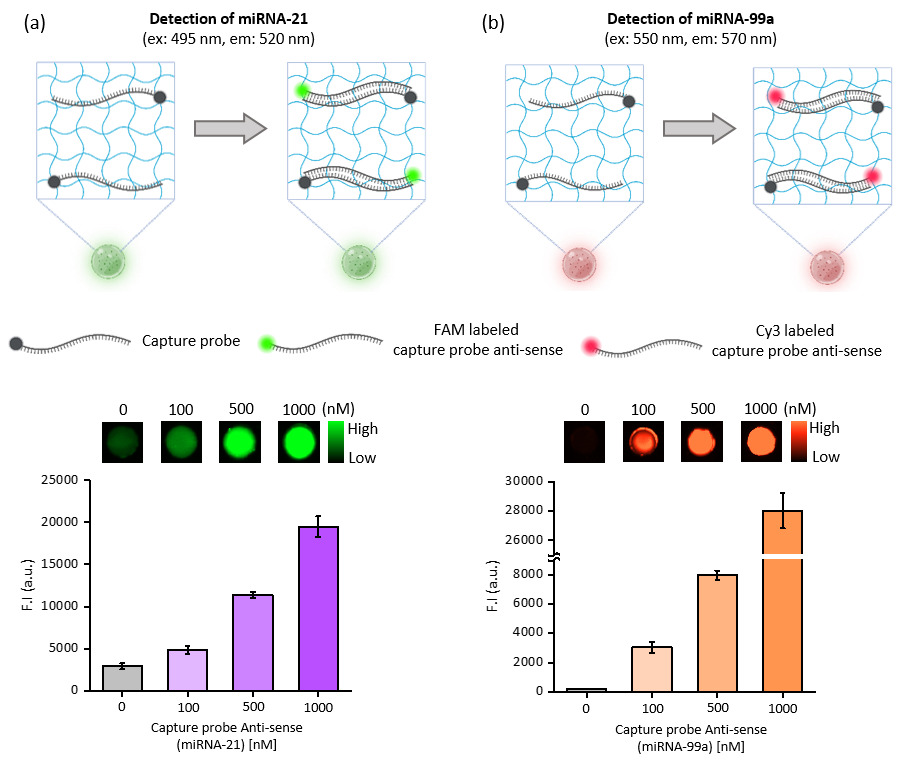


**Figure S7**. Confirmation of capture probe immobilization within the hydrogel. (a,b) Optimization of fluorescence intensity within the hydrogel by using varying concentrations of anti-capture probes targeting (a) miRNA-21 and (b) miRNA-99a. Data represent mean ± SD for three independent experiments.


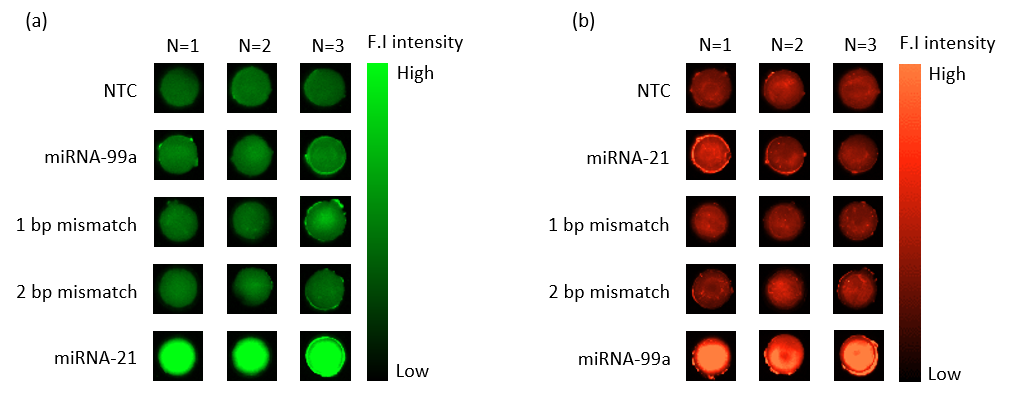


**Figure S8**. Hydrogel images depicting specific detection of target miRNA in the NACH assay. (a) Specificity for (a) miRNA-21 (λ_ex_: 450; λ_em_: 520) and (b) miRNA-99a (λ_ex_: 550; λ_em_: 570) in the NACH assay, respectively.


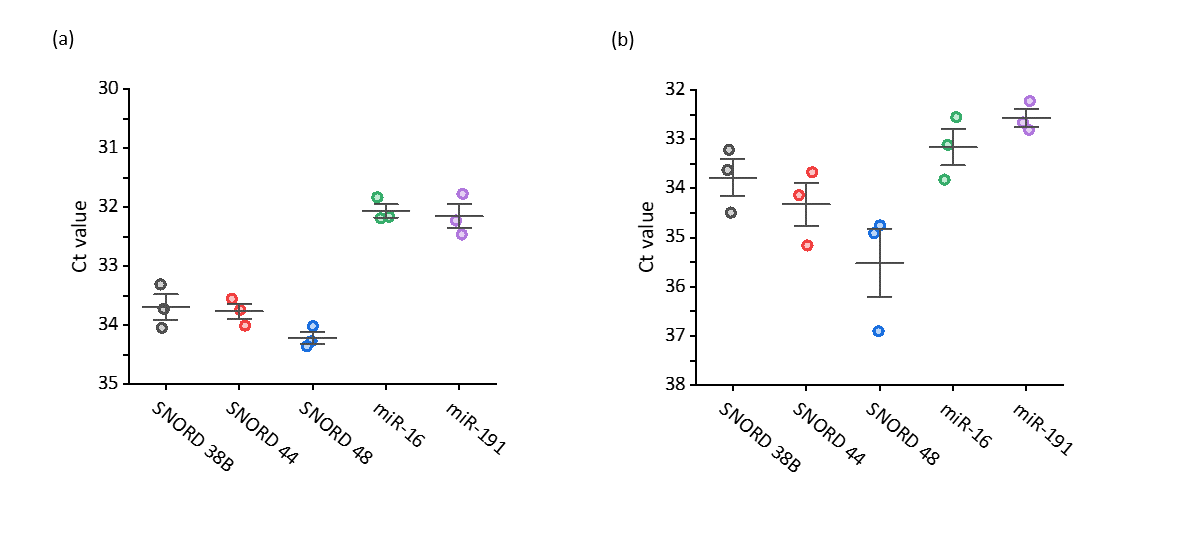
**Figure S9**. Analysis of qRT-PCR data for selecting reference genes in gastric cancer cell lines. Analysis of reference gene expression levels in the (a) SNU-484 and (b) Hs 746T cell lines.


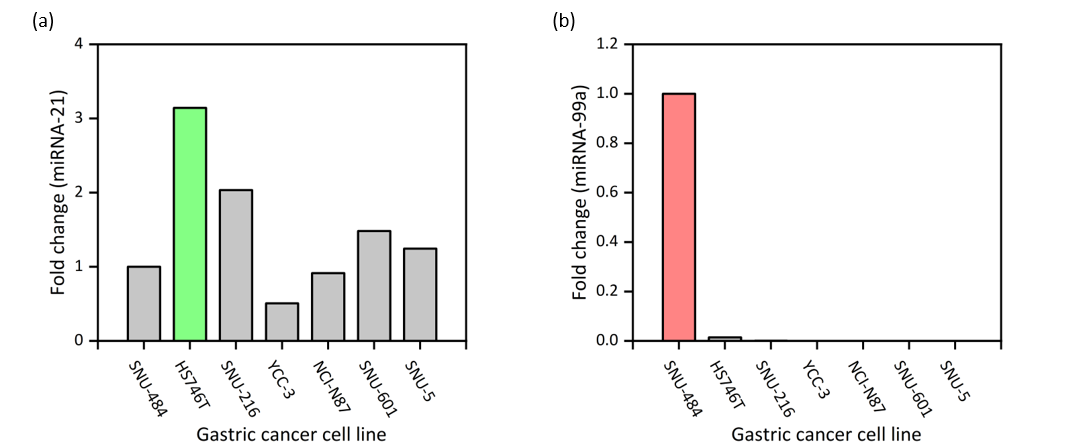


**Figure S10**. Analysis of the expression levels of (a) miRNA-21 and (b) miRNA-99a using qRT-PCR in gastric cancer cell lines.


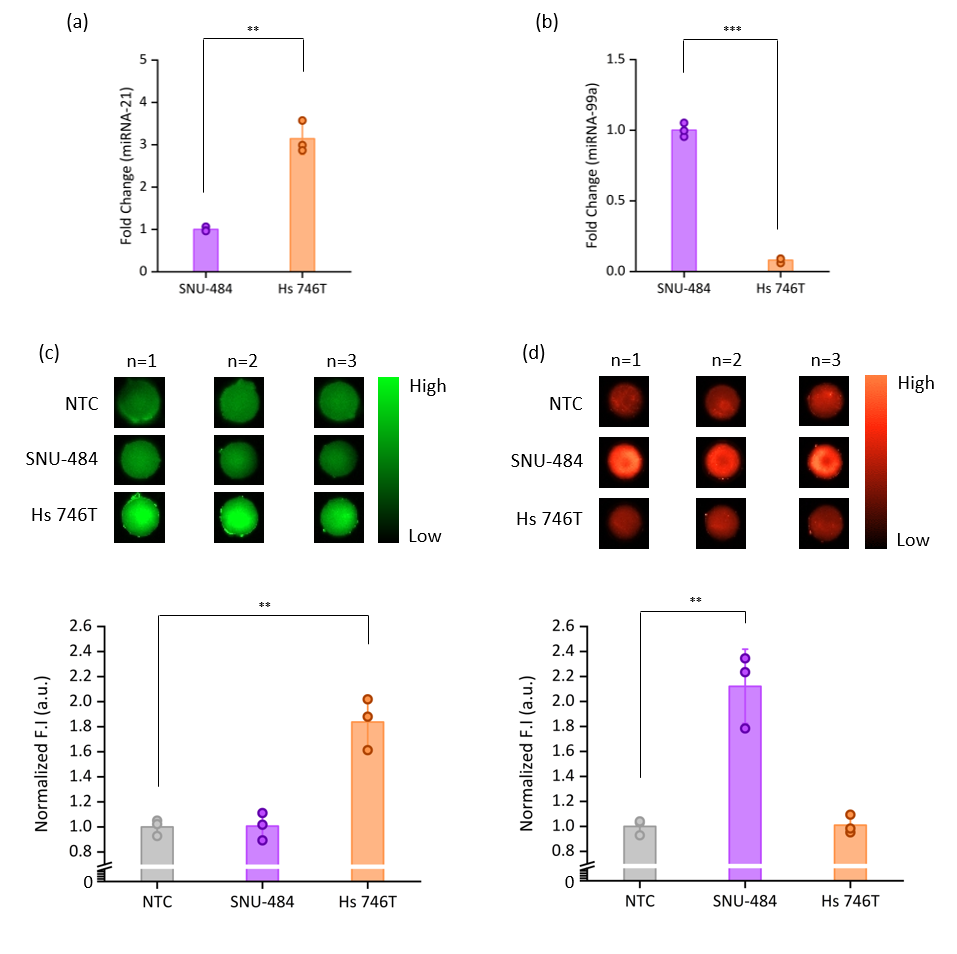


**Figure S11**. Evaluation of the cellular RNA detection performance of the NACH assay. (a,b) Quantitative real-time PCR (qRT-PCR) analysis of (a) miRNA-21 and (b) miRNA-99a expression levels in SNU-484 and Hs 746T gastric cancer (GC) cell lines. The expression levels of miRNA were normalized to those of miRNA-16 and miRNA-191. (c,d) Fluorescence and associated intensity corresponding to (c) miRNA-21 and (d) miRNA-99a upon using total cellular RNA (50 ng/hydrogel) isolated from SNU-484 and Hs 746T GC cells (n = 3). Data represent mean ± SD for three independent experiments. ****p* < 0.0005, ***p* < 0.005.


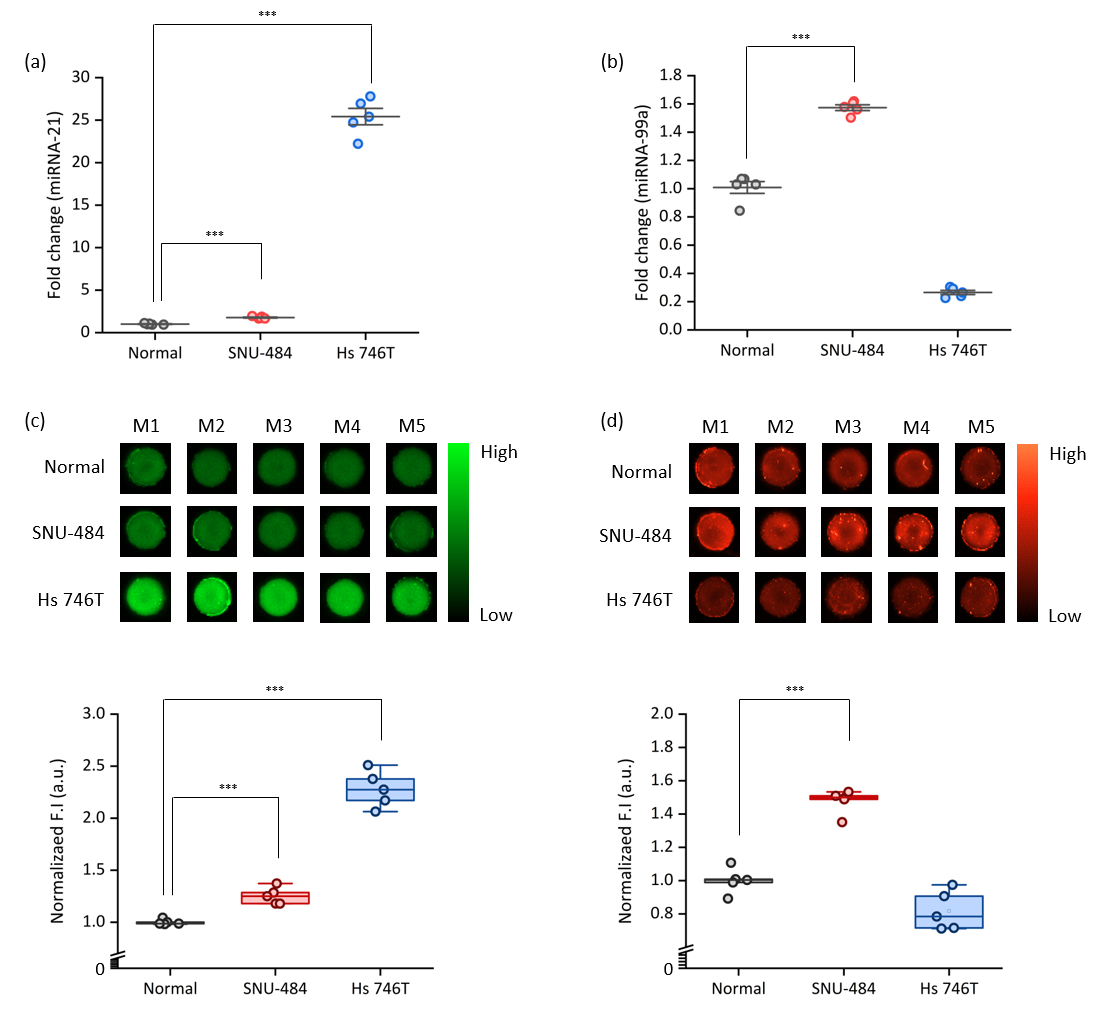


**Figure S12**. Evaluation of the performance of the NACH assay with respect to the detection of miRNA extracted from mouse tumor tissue. Total miRNA was isolated from the tumor tissue of three different mouse groups (Normal, SNU-484, Hs 746T). (a,b) qRT-PCR analysis comparing the expression levels of (a) miRNA-21 and (b) miRNA-99a, with the miRNA expression levels normalized to miRNA-16 and miRNA-191. (c,d) Fluorescence images and associated intensity corresponding to (c) miRNA-21 and (d) miRNA-99a upon using total miRNA (100 ng/hydrogel) isolated from mouse tumor tissue (n=5). ****p* < 0.0005.


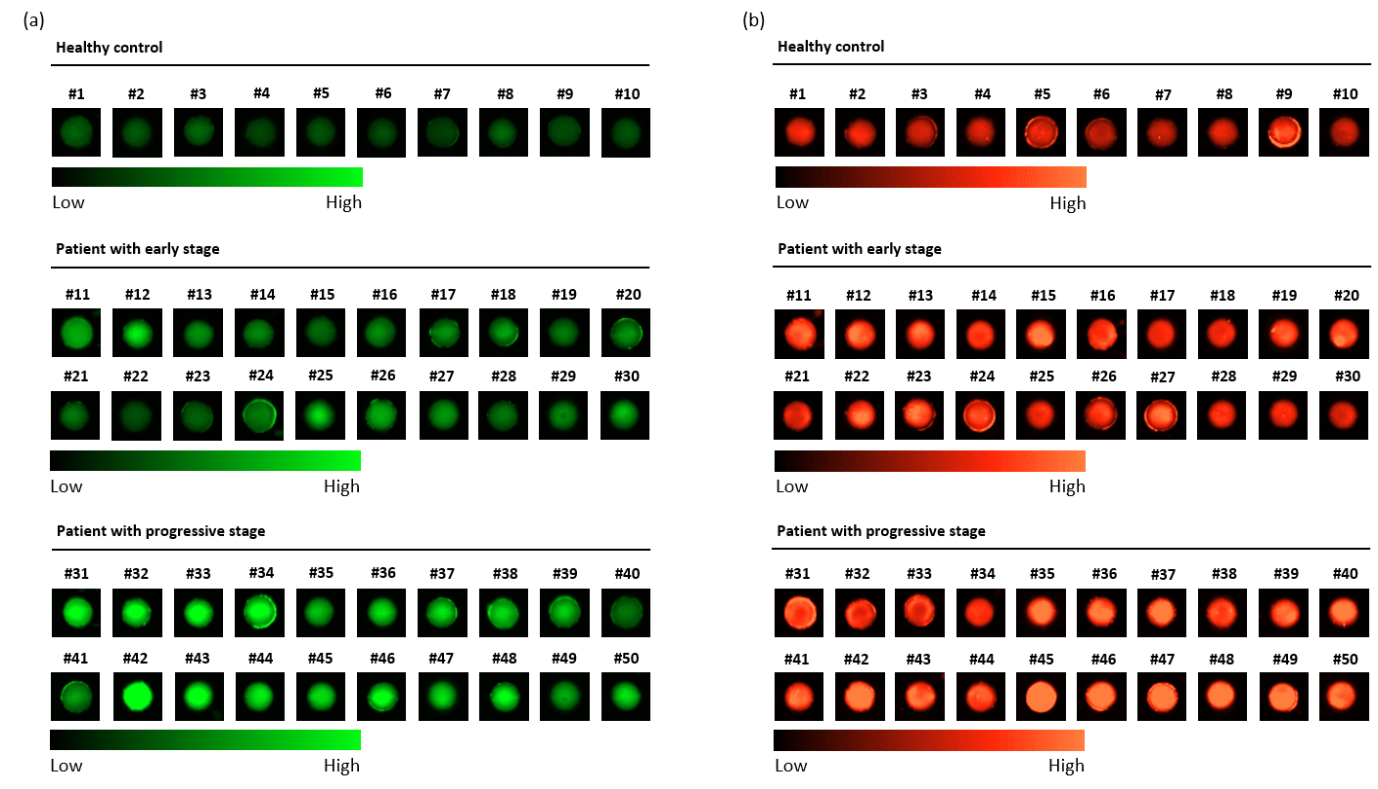


**Figure S13**. Hydrogel images when using clinical samples in the NACH assay. (a) Hydrogel images of miRNA-21 in the NACH assay according to patients. (b) Hydrogel images of miRNA-99a in the NACH assay according to patients.


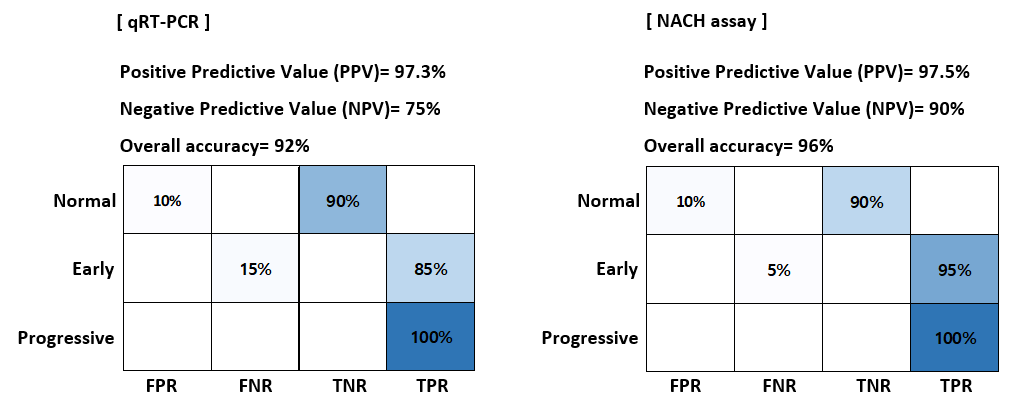


**Figure S14.** Statistical data on the sensitivity, specificity, and predictive value of qRT-PCR and NACH assay.


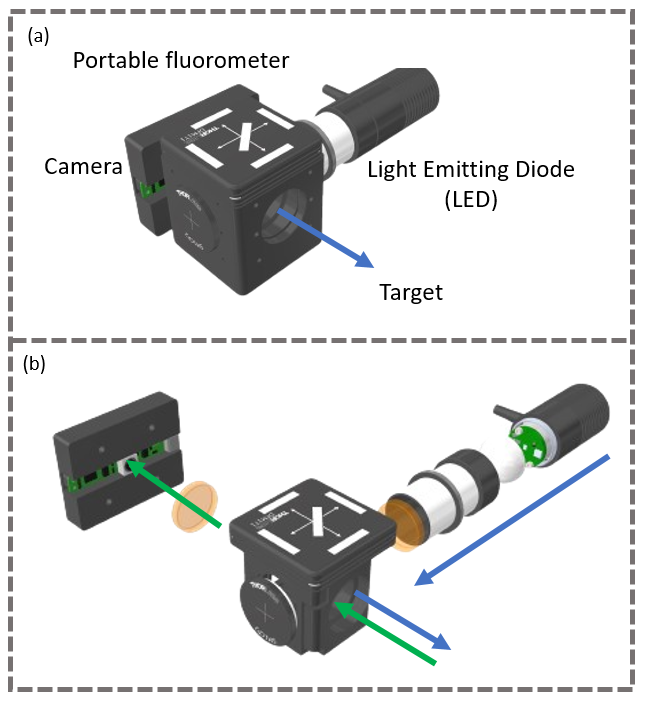


**Figure S15**. Schematic illustration of a portable fluorometer. (a) External schematic of the portable fluorometer (b) Internal schematic of the portable fluorometer. Indication of the direction of the blue (excitation) and green (emission) lights.


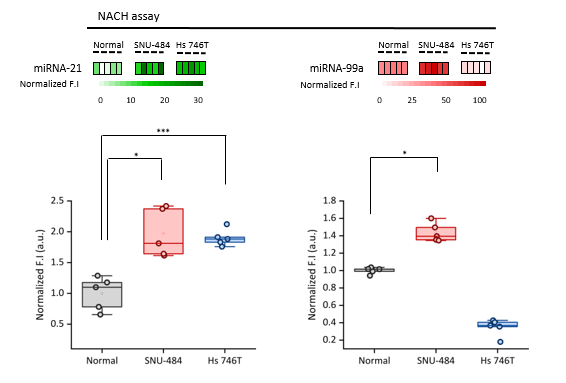


**Figure S16**. Evaluation of the use of mouse plasma in the NACH assay using a portable fluorometer. Heatmap showing the fluorescence intensity corresponding to miRNA-21 (green) and miRNA-99a (red) in exosome samples obtained from the plasma of mice independently injected with SNU-484 and Hs 746T cells (n = 5) in the NACH assay using a portable fluorometer. The fluoresecence intensity represents miRNA-21 (green) and miRNA-99a (red) obtained upon using total miRNA extracted from the plasma of mice independently injected with SNU-484 and Hs 746T cells. ****p* < 0.0005, **p* < 0.05.

**Table S1**. Sequences of NACH assay probes.

| Name | Sequence (5′ → 3′) | Modification |
| --- | --- | --- |
| Target DNA (miRNA-21) | TAG CTT ATC AGA CTG ATG TTG A | - |
| Target DNA (miRNA-99a) | AAC CCG TAG ATC CGA TCT TGT G | - |
| Padlock probe (miRNA-21) | GAT GTA AAT CAC AAA GAA GGG GAA TAC AAC AAC AG T CAA CAT CAG T | 5′ – Phosphate |
| Padlock probe (miRNA-99a) | GAT GTA AAT CAC AAA GAA GGG GAA TAC AAC AAC AG C ACA AGA TCG G | 5′ – Phosphate |
| Capture probe  (miRNA-21) | CTG ATA AGC TAT TTT TTT TTT TTT TT | 3′ – Acrylamide |
| Capture probe (miRNA-99a) | ATC TAC GGG TTT TTT TTT TTT TTT TT | 3′ – Acrylamide |
| Reporter probe  (miRNA-21) | (FAM) GGA ATA CAA CAA CAG | 5′ – FAM |
| Reporter probe  (miRNA-99a) | (Cyanine 3) AGG GGA ATA CAA CAA | 5′ – Cyanine 3 |

**Table S2**. Comparison of hydrogel-based nucleic acid detection methods.

| Method | Target | Mechanism | LOD | Properties | Ref |
| --- | --- | --- | --- | --- | --- |
| RCA-based assay (Rolling circle amplification) | miRNA-21, Let7a, miRNA-19b | RCA + Hydrogel | 2.3 zmol (miRNA-21)  44 zmol (Let7a)  17 zmol (miRNA-19b) | - Multi-step reaction (time intensive)  - Multi-step reaction  - Temperature cycling reaction | [1] |
| CLAMP (Cas-loaded annotated micro-particles) assay | HPV (dsDNA) | CRISPR Cas12a + Hydrogel | 3 pM | - Low sensitivity  - Multi-step reaction | [2] |
| Hydrogel LAMP system | *E. coli* (dsDNA) | LAMP + Hydrogel | Single molecule | - Multi-step reaction  - Request of specific equipment | [3] |
| Hydrogel-based HCR (hybridization chain reaction) | miRNA-6090, miRNA-3665 | HCR + Hydrogel | 1 amol (miRNA-6090) 10 amol (miRNA-3665) | - Low sensitivity  - Temperature cycling reaction  - Multi-step reaction | [4] |
| Hydrogel-based LDR (ligation detection reaction)/RCA (rolling circle amplification) assay | Ginseng (dsDNA) | LDR/RCA + Hydrogel | 0.75 pM | - Temperature cycling reaction  - Multi-step reaction | [5] |
| NACH (nucleic acid amplification circuit-based hydrogel) assay | **miRNA-21, miRNA-99a** | **RCA + Hydrogel** | **1 fM (miRNA-21)**  **1 fM (miRNA-99a)** | **- One-step reaction**  **- Isothermal reaction**  **- Portable fluorescence module applicable** | **NACH assay** |

**Table S3. Comparison of biosensor methods for miRNA detection**

| Method | Target | LOD | Disease | Detection time | Validation | Reference |
| --- | --- | --- | --- | --- | --- | --- |
| SORTER | miR-21, miR-222, miR-1290, miR-221, miR-10b, miR-182 (Exosome) | 1.2×10^5^ particles/µl (Exosome) | Prostate cancer | 2 hours | *Clinical* level | [6] |
| TDN | miR-21  (Exosome) | 34 aM | Breast cancer | ~ 3 hours | *Clinical* level | [7] |
| MERCA | Let-7a, miR-21  (Exosome) | 10 zmol | Lung cancer | > 4 hours | *In vitro* level | [8] |
| HCR Electroanalytical Assay | miR-122  (Exosome) | 53 aM | Breast cancer | > 3 hours | *In vitro* level | [9] |
| Enzymes-assisted dual-signal amplification | miR-21  (Exosome) | 0.34 fM | Gastric cancer | > 8 hours | *Clinical* level | [10] |
| hydrogel-based hybridization  chain reaction | miR-6090, miR-3665  (Exosome) | miR-6090 (1-10 amol)  miR-3665 (10-100 amol) | Prostate cancer | > 3 hours | *Clinical* level | [11] |
| SERS biosensor | miR-10a, miR-21  (Exosome) | 10 aM | Prostate cancer | > 12 hours | *Clinical* level | [12] |
| EXTRA-CRISPR assay | miR-21, miR-196b  miR-451a, miR-1246  (Extracellular vesicles) | 1.64 fM | pancreatic cancer | >2 hours | *Clinical* level | [13] |
| NACH assay | **miR-21,**  **miR-99a** | **1.0 fM** | **Gastric cancer** | **2 hours** | ***Clinical* level** | **Our work** |

**Table S4**. Information corresponding to healthy individuals and gastric cancer patients.

|  | Sex | Age (years) | Stage |
| --- | --- | --- | --- |
| #1 | Female | 45 | - |
| #2 | Male | 73 | - |
| #3 | Female | 59 | - |
| #4 | Female | 65 | - |
| #5 | Male | 66 | - |
| #6 | Male | 52 | - |
| #7 | Female | 78 | - |
| #8 | Male | 72 | - |
| #9 | Female | 56 | - |
| #10 | Male | 81 | - |
| #11 | Male | 45 | I |
| #12 | Female | 73 | I |
| #13 | Male | 59 | I |
| #14 | Male | 65 | I |
| #15 | Male | 66 | I |
| #16 | Male | 52 | I |
| #17 | Male | 78 | I |
| #18 | Female | 72 | I |
| #19 | Male | 56 | I |
| #20 | Male | 81 | I |
| #21 | Female | 57 | I |
| #22 | Male | 74 | I |
| #23 | Female | 62 | I |
| #24 | Male | 87 | I |
| #25 | Female | 65 | I |
| #26 | Female | 45 | I |
| #27 | Male | 57 | I |
| #28 | Male | 55 | I |
| #29 | Male | 66 | I |
| #30 | Male | 70 | I |
| #31 | Male | 58 | II-III |
| #32 | Female | 77 | II-III |
| #33 | Male | 66 | II-III |
| #34 | Male | 51 | II-III |
| #35 | Male | 68 | II-III |
| #36 | Male | 62 | II-III |
| #37 | Male | 77 | II-III |
| #38 | Female | 49 | II-III |
| #39 | Male | 67 | II-III |
| #40 | Male | 73 | II-III |
| #41 | Male | 46 | II-III |
| #42 | Male | 45 | II-III |
| #43 | Male | 74 | II-III |
| #44 | Male | 59 | II-III |
| #45 | Male | 73 | II-III |
| #46 | Male | 72 | II-III |
| #47 | Male | 89 | II-III |
| #48 | Male | 59 | II-III |
| #49 | Female | 77 | II-III |
| #50 | Male | 82 | II-III |

**References**

[1] D.A. Sulaiman, N. Juthani, P.S. Doyle, *Adv Healthc Mater* **2022**, 11, e2102332. DOI: 10.1002/adhm.202102332.

[2] Y.H. Roh, C.Y. Lee, S. Lee, H. Kim, A. Ly, C.M. Castro, *Adv Sci* **2023**, 10, e2206872. DOI: 10.1002/advs.202206872.

[3] C. Yi, Z. Luo, Y. Lu, T. Belwal, X. Pan, X. Lin, *Biosens Bioelectron* **2021**, 184, 113199. DOI: 10.1016/j.bios.2021.113199.

[4] J. Kim, J.S. Shim, B.H. Han, H.J. Kim, J. Park, I.-J. Cho, S.G. Kang, J.Y. Kang, K.W Bong, N. Choi, *Biosens Bioelectron* **2021**, 192, 113504. DOI: 10.1016/j.bios.2021.113504.

[5] S.J. Mun, W. Jang, H.-S. Park, Y.J. Lim, T.-J. Yang, K.W. Bong, *Biosens Bioelectron* **2023**, 241, 115670. DOI: 10.1016/j.bios.2023.115670.

[6] Y. Lei, X. Fei, Y. Ding, J. Zhang, G. Zhang, L. Dong, J. Song, Y. Zhuo, W. Xue, P. Zhang, C. Yang, *Sci. Adv* **2023**, 9, eadi1556. DOI: 10.1126/sciadv.adi1556.

[7] N. Liu, H. Lu, L. Liu, W. Ni, Q. Yao, G.J. Zhang, F. Yang, *Anal. Chem* **2021**, 93, 5917-5923. https://doi.org/10.1021/acs.analchem.1c00295.

[8] H. Cao, X. Zhou, Y. Zeng, *Sens Actuators B Chem* **2019**, 15, 447-457. https://doi.org/10.1016/j.snb.2018.09.121.

[9] Q. Guo, Y. Yu, H. Zhang, C. Cai, Q. Shen, *Anal. Chem* **2020**, 92, 5302-5310. https://doi.org/10.1021/acs.analchem.9b05849.

[10] Y. Xia, Z. Huang, T. Chen, L. Xu, G. Zhu, W. Chen, G. Chen, S. Wu, J. Lan, X. Lin, J. Chen, *Biosens Bioelectron* **2022**, 209, 114259. https://doi.org/10.1016/j.bios.2022.114259.

[11] J. Kim, J.S. Shim, B.H. Han, H.J. Kim, J. Park, I.J. Cho, S.G. Kang, J.Y. Kang, K.W. Bong, N. Choi, *Biosens Bioelectron* **2021**, 192, 113504. https://doi.org/10.1016/j.bios.2021.113504.

[12] W.H. Kim, J.U. Lee, M.J. Jeon, K.H. Park, S.J. Sim, *Biosens Bioelectron* **2022**, 205, 114116. https://doi.org/10.1016/j.bios.2022.114116.

[13] H. Yan, Y. Wen, Z. Tian, N. Hart, S. Hang, S.J. Hughes, Y. Zeng, *Nat. Biomed. Eng* **2023**, 7, 1583-1601. https://doi.org/10.1038/s41551-023-01033-1.
